# The ESCRT-0 subcomplex component Hrs/Hgs is a master regulator of myogenesis via modulation of signaling and degradation pathways

**DOI:** 10.1186/s12915-021-01091-4

**Published:** 2021-07-30

**Authors:** L. Coudert, A. Osseni, Y. G. Gangloff, L. Schaeffer, P. Leblanc

**Affiliations:** Institut NeuroMyoGène, CNRS UMR5310, INSERM U1217, Faculté de Médecine Rockefeller, Université Claude Bernard Lyon, 8 avenue Rockefeller, 69373, 09 Lyon, Cedex France

**Keywords:** ESCRT, Hrs/Hgs, Myogenesis, Myoblast/Myotube, Differentiation, Skeletal muscle, Endosomes, Autophagy, Signaling

## Abstract

**Background:**

Myogenesis is a highly regulated process ending with the formation of myotubes, the precursors of skeletal muscle fibers. Differentiation of myoblasts into myotubes is controlled by myogenic regulatory factors (MRFs) that act as terminal effectors of signaling cascades involved in the temporal and spatial regulation of muscle development. Such signaling cascades converge and are controlled at the level of intracellular trafficking, but the mechanisms by which myogenesis is regulated by the endosomal machinery and trafficking is largely unexplored. The Endosomal Sorting Complex Required for Transport (ESCRT) machinery composed of four complexes ESCRT-0 to ESCRT-III regulates the biogenesis and trafficking of endosomes as well as the associated signaling and degradation pathways. Here, we investigate its role in regulating myogenesis.

**Results:**

We uncovered a new function of the ESCRT-0 hepatocyte growth factor-regulated tyrosine kinase substrate Hrs/Hgs component in the regulation of myogenesis. Hrs depletion strongly impairs the differentiation of murine and human myoblasts. In the C2C12 murine myogenic cell line, inhibition of differentiation was attributed to impaired MRF in the early steps of differentiation. This alteration is associated with an upregulation of the MEK/ERK signaling pathway and a downregulation of the Akt2 signaling both leading to the inhibition of differentiation. The myogenic repressors FOXO1 as well as GSK3β were also found to be both activated when Hrs was absent. Inhibition of the MEK/ERK pathway or of GSK3β by the U0126 or azakenpaullone compounds respectively significantly restores the impaired differentiation observed in Hrs-depleted cells. In addition, functional autophagy that is required for myogenesis was also found to be strongly inhibited.

**Conclusions:**

We show for the first time that Hrs/Hgs is a master regulator that modulates myogenesis at different levels through the control of trafficking, signaling, and degradation pathways.

**Supplementary Information:**

The online version contains supplementary material available at 10.1186/s12915-021-01091-4.

## Background

Differentiation of myoblasts into myotubes is a tightly concerted and highly regulated process that is initiated by the binding of extracellular ligands to their surface membrane receptors and by cell-cell contacts that collectively engage different downstream signaling cascades that ultimately lead to the expression of myogenic regulatory/transcription factors (MRFs) such as MyoD and myogenin [1, 2]. MRFs act synergistically with the myocyte enhancer factor 2 (MEF2) to drive the successive steps of the myogenic program through which myoblasts become post mitotic myocytes that fuse to form multinucleated myotubes further acquiring contractile capabilities [2–5].

The tight control of cellular responsiveness to extracellular ligands involves the endocytosis of ligand-activated receptors and their redirection to endosomes or endo-lysosomal compartments. In this context, early endosomes and multivesicular bodies (MVBs) are crucial sorting stations that play a key role in the control of the duration of the cellular responsiveness to given ligands by orienting endocytosed complexes to various pathways such as degradation, recycling, scaffolding, sequestration, or catalysis (reviewed in [6]). The control of myogenesis by the endocytic machinery and by endosome trafficking is still poorly investigated but is progressively gaining interest [7–15].

The Endosomal Sorting Complex Required for Transport (ESCRT) is an essential machinery involved in important cellular processes. It is composed of approximatively 20 proteins assembled into four distinct heteromeric protein subcomplexes named ESCRT-0, I, II, and III according to their chronological involvement in sorting ubiquitinated cargos into MVBs (reviewed in [16, 17]). The Human HRS/HGS or murine Hrs/Hgs (HGS/Hgs for hepatocyte growth factor-regulated tyrosine kinase substrate)-ESCRT-0 is a key actor of endosomal sorting. First, HRS directly binds ubiquitinated proteins and participates to the sorting of ubiquitinated cargos into clathrin-coated microdomains on early endosomes. Second, HRS recruits the TSG101 ESCRT-I component, and beyond that, it orchestrates many events surrounding MVB biogenesis. Many studies have identified HRS functions unrelated to the recruitment of TSG101 and to the ESCRT pathway. For example, HRS specifically regulates endosomal cholesterol trafficking independently of other ESCRT proteins including ESCRT-I TSG101 [18]. Along this line, HRS, but not TSG101, promotes actin-mediated endosomal recycling of receptors back to the plasma membrane [19–21]. Most importantly, HRS modulates different signaling pathways through the regulation of the trafficking and degradation of membrane receptors. For example, the silencing of HRS prevents the degradation of the epidermal growth factor receptor (EGFR) through the lysosomal pathway, leading to its accumulation into the early endosomes and thus upregulating EGFR downstream signaling [22–25]. Conversely, HRS is involved in the stimulation of the Wnt signaling pathway by promoting sequestration of the Glycogen Synthase Kinase 3 beta (GSK3β) into early endosomes, thereby preventing the phosphorylation and the degradation of β-catenin [26, 27]. Finally, different studies revealed that HRS can modulate the autophagic flux [28–30].

Altogether, HRS modulates many cellular processes known to be involved in myogenesis but until today no study investigated the role of HRS/HGS during this process.

First, we show that Hrs expression and distribution were sharply regulated during myoblast differentiation. Second, we observed that HRS depletion in murine and human muscle cells strongly impaired myoblasts differentiation. Third, in the C2C12 model, we found that MyoD level in the nucleus was decreased and the upregulation of MEF2 expression as well as myogenin were prevented, consistently with the downregulation of Akt2 global activity and the upregulation of the early myogenesis repressor FOXO1. Fourth, upstream of MRFs and MEF2, the myogenic inhibitory MEK/ERK pathway was activated in the early steps of differentiation upon Hrs depletion leading to the negative regulation of the myogenesis process. Pharmacological inhibition of this signaling pathway significantly restores differentiation of Hrs-depleted myoblasts highlighting the essential role of this signaling pathway. Fifth, our data also revealed that the trafficking and the degradation of EGFR, a known regulator of MEK/ERK, were impaired in absence of Hrs, leading to the upregulation of its activity. Sixth, our data also showed that activity of GSK3β, a repressor of myogenesis, was upregulated upon Hrs silencing and that its inhibition partially restores the differentiation process. Last,we found that functional autophagy that isrequired for myogenesis was also strongly inhibited in absence of Hrs.

Altogether, our results identify, for the first time, Hrs as a master regulator of myogenesis.

## Results

### Hrs expression and distribution are modulated during myoblast differentiation

**Fig. 1:**
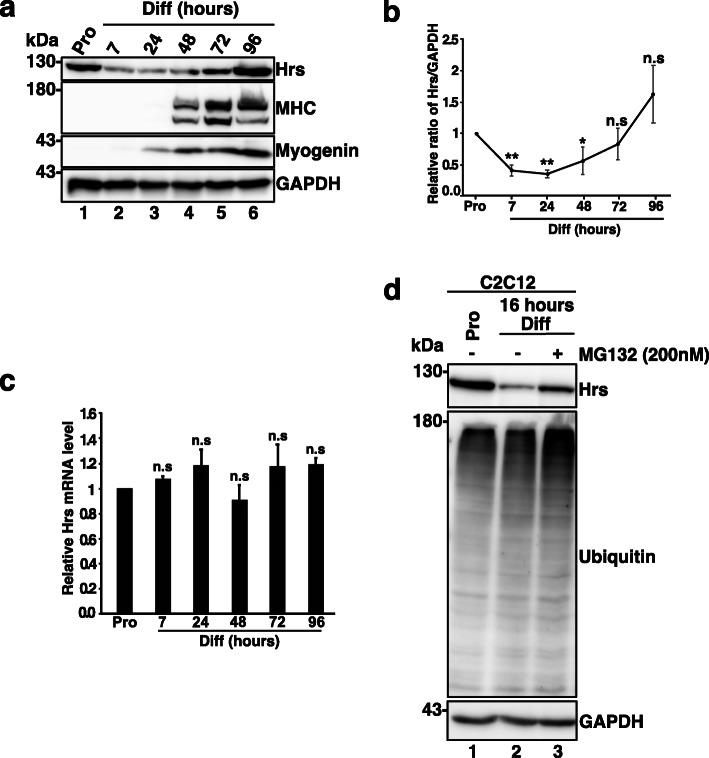
Hrs is dynamically expressed and distributed during myogenesis in C2C12 and primary muscle cells. **a** Representative Western blotting of myoblast C2C12 cell extracts in Pro status (lane 1) and at 7, 24, 48, 72, and 96 h of differentiation (lanes 2–6) and probed with anti-MHC, -Myogenin as makers of differentiation, and anti-HRS antibody. **b** Quantification of the Hrs protein level from experiments as presented in **a**. Data are presented as ratio of Hrs/GAPDH and normalized to the Pro status starting point. Data represent mean +/− SEM. *n* = 8 experiments. Significance was assessed using a Kruskal–Wallis test; **p < 0.01; ***p < 0.001. **c** qRT-PCR of Hrs-mRNA from C2C12 cells collected in Pro and at 7, 24, 48, 72, and 96 h of differentiation. Data represent mean +/− SEM, *n* = 3 experiments. Significance was assessed using a Kruskal–Wallis test; ns not significant. **d** Representative Western blotting of C2C12 in Pro or at 16 h of differentiation treated with DMSO or 200 nM MG132 proteasome inhibitor. Membrane was probed with anti-HRS, -ubiquitin. GAPDH was used as loading control

Hrs levels and distribution were evaluated in C2C12 myoblasts cultured in proliferation medium (Pro) and switched to differentiation medium (Diff) to initiate their differentiation into myotubes. Efficient differentiation was evaluated by myosin heavy chain (MHC) expression (lanes 4–6). Hrs protein level was high in Pro and strongly decreased when C2C12 cells were shifted to Diff (compare lanes 1 and 2 and quantifications in Fig. 1a,b). Hrs level then progressively increased again between 48 and 96 h (lanes 4–6 in Fig. 1a and quantifications in Fig. 1b). Quantitative RT-PCR (qRT-PCR) analyses revealed no significant variation in Hrs-mRNA level (Fig. 1c). Recent studies revealed that Hrs can be degraded by the proteasome upon starvation [31, 32]. Treatment of C2C12 cells with the MG132 proteasome inhibitor significantly restored Hrs protein level at 16 h of differentiation compared to DMSO-treated cells (Fig. 1d). These results indicate that transient decrease of Hrs level at the onset of differentiation is due to proteasomal degradation.

Interestingly, Hrs upregulation appears to follow the increase of myogenin expression (see lane 6 in Fig. 1a) possibly indicating a role of Hrs in myogenesis.

**Fig. 2:**
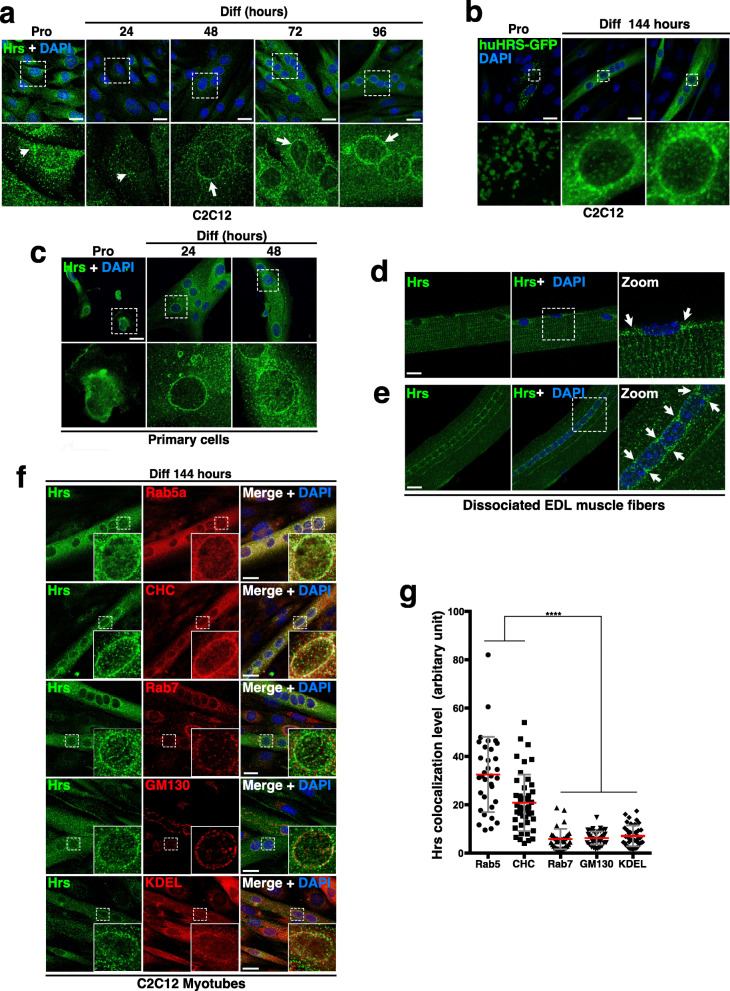
Significant perinuclear colocalization of Hrs with Rab5a and Clathrin heavy chain (CHC) in differentiated myotubes. **a** Representative immunofluorescence images of Hrs distribution in C2C12 in Pro or during differentiation at the indicated time points (in hours). Cells were stained with the anti-HRS (green) and DAPI (blue) for nuclear staining. White dotted squares represent a focus of the selected region (lower panel). White arrowheads/arrows show cytoplasmic/perinuclear Hrs distribution respectively during the course of myogenesis. **b** Representative immunofluorescence images of C2C12 transfected with the human GFP-HRS (huHRS-GFP) encoding construct in Pro or at 144 h of differentiation. huHRS-GFP signal (green) and DAPI (blue). White dotted squares represent a focus of the selected region (lower panel). **c** Representative immunofluorescence images of Hrs distribution in primary murine muscle cells in Pro or at 24 and 48 h of differentiation. Cells were stained with anti-HRS (green) and DAPI (blue). White dotted squares represent a focus of the selected region (lower panel). **d,e** Representative immunofluorescence images of dissociated EDL muscle fibers stained with anti-HRS (green) and DAPI (blue). White dotted squares represent a focus of the selected region (right panel). Perinuclear Hrs (white arrows). **f** Representative immunofluorescence images of Hrs distribution in C2C12 at 144 h of differentiation. Cells were probed with the anti-HRS (green) and the anti-Rab5a for EE or anti-CHC; anti-Rab7; -KDEL and -GM130 for LE; ER or Golgi respectively (in red). Note the accumulation of Hrs around and at the poles of nuclei. White dotted squares represent a focus of the selected regions (see insets). **g** Quantifications of colocalization from experiments like in **f** have been made by creating binary mask between merge channel of Hrs and Rab5a, CHC, Rab7, KDEL, or GM130 signals. Quantification of the signals obtained around nuclei is presented as plot-graphs where one dot corresponds to the signal for one nucleus. Data represent mean +/− SD, *n* = 33–45 nuclei for each group. *n* = 3. Significance was assessed using a Mann–Whitney *U* test (****p < 0.0001). Scale bars, 20 μm

We next investigated the cellular distribution of Hrs during C2C12 differentiation. Hrs immunolabeling showed a bi-modal distribution with punctuated cytoplasmic distribution in myoblasts (see white arrowheads in Fig. 2a), and at the nuclear periphery from 48 h in Diff (see white arrows in Fig. 2a). Hrs perinuclear distribution in myotubes was confirmed in C2C12 transfected with a human GFP-HRS construct whereas it displays a punctuated cytoplasmic distribution in transfected myoblasts (Fig. 2b). Immunofluorescence experiments in murine primary muscle cells confirmed the ring perinuclear localization of Hrs in differentiated myotubes (Fig. 2c). Furthermore, accumulation of Hrs protein was also observed at the nuclear poles and around nuclei (see white arrows in Fig. 2d) in dissociated muscle fibers of the extensor digitorum longus muscle (EDL) from 3-month-old mice. In regenerating muscle fibers, which typically display aligned and centrally located nuclei, a strong and specific accumulation of perinuclear Hrs was also detected (see white arrows in Fig. 2e).

Colocalization experiments with Rab5a or Rab7 to label early (EE) and late (LE) endosomes respectively, the GM130 Golgi marker, the KDEL endoplasmic reticulum (ER) marker, or clathrin heavy chain (CHC) to visualize clathrin-coated vesicles revealed that perinuclear Hrs colocalized with Rab5a positive early endosomes in myotubes (Fig. 2f,g). As expected, Hrs also colocalized with CHC, a well characterized binding partner of Hrs [33, 34]. Conversely, Hrs did not show significant colocalization with LE, ER, or Golgi apparatus (Fig. 2f,g).

Altogether, our results revealed a modulation of Hrs protein level and localization during the course of differentiation.

To identify possible co-regulations, Tsg101 (Tumor Susceptibility gene 101) ESCRT-I protein expression was investigated. In contrast to Hrs, Tsg101 did not display a bi-modal expression but a progressive increase during the differentiation process (Additional file 1 Fig. S1a,b).

Therefore, although Hrs and Tsg101 belong to the same ESCRT machinery, their regulation during myogenesis is different.

### Silencing of Hrs strongly affects myogenesis

**Fig. 3:**
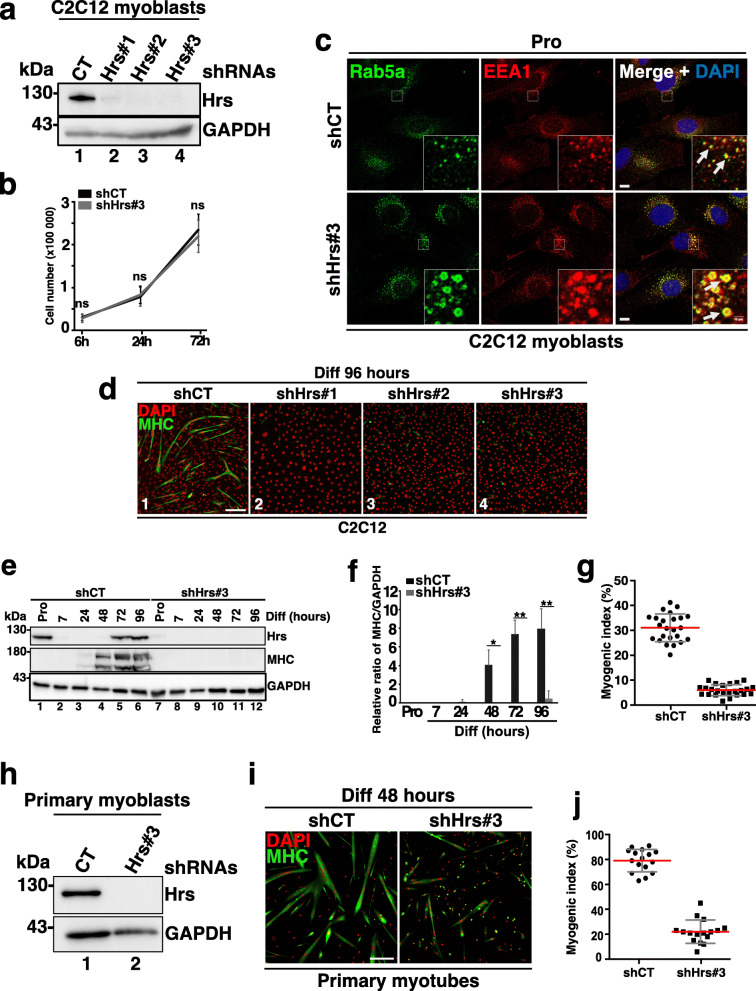
Hrs silencing inhibits myoblast to myotube differentiation. **a** Representative Western blotting of C2C12 transduced with HIV-1 lentivectors shCT (lane 1) or shHrs-mRNA shHrs#1 (lane 2), #2 (lane 3), #3 (lane 4) and probed with the anti-HRS and anti-GAPDH. **b** Quantification of shCT and shHRS#3 C2C12 cell number at 6, 24, or 72 h. Data represent mean +/− SEM, *n* = 3 experiments. Significance was assessed using two-tailed Mann–Whitney *U* test. ns not significant. **c** Representative immunofluorescence images of shCT and shHrs#3 C2C12 myoblasts and probed with anti-Lamp1 (green) for LE/LYS or anti-EEA1 (red) for EE and DAPI (blue). White dotted squares represent a focus of the selected regions (see insets). Scale bar, 20 μm. **d** Representative immunofluorescence images of shCT and shHrs#1-3 C2C12 at 96 h of differentiation and probed with the anti-MHC (green) as a marker of myotubes and DAPI (red) for nuclear staining. Scale bar, 200 μm. **e** Representative Western blotting of shCT and shHrs#3 C2C12 myoblasts at the Pro status (lanes 1,7) or during the differentiation 7–96 h (lanes 2–6 and 8–12) and probed with the anti-HRS and -MHC. GAPDH was used as a loading control. **f** Quantification of the MHC protein level present in experiments like in **e**. Data are presented as ratio of MHC/GAPDH and normalized to the Pro starting point condition. Data represent mean +/− SEM, *n* = 3 experiments. Significance was assessed using a two-way ANOVA test; *p < 0.05, **p < 0.01. **g** Quantification of the myogenic index corresponding to the number of nuclei present in MHC-positive cells as shown in **d**. Data represent mean +/− SEM, each dot represents one image, *n* = 24 images from two independent experiments. **h** Representative Western blotting of primary muscle cells transduced with the lentivector shCT (lane 1) or shHrs#3 (lane 2) and probed with the anti-HRS. GAPDH was used as a loading control. **i** Representative immunofluorescence images of shCT and shHrs#3 primary muscle cells at 48 h of differentiation and probed with the anti-MHC (green) and DAPI (red). Scale bar, 200 μm. **j** Quantification of the myogenic index corresponding to the number of nuclei present in MHC-positive cells as shown in **i**. Data represent mean +/− SEM, each dot represents one image, *n* = 16 images from two independent experiments

To explore the role of Hrs during myogenesis, we assessed the impact of Hrs silencing on differentiation. Three independent lentiviral vectors encoding Hrs-shRNAs (shRNAs #1 to #3) and a non-specific shRNA target (shCT) were used to transduce C2C12 myoblasts. The three shRNAs efficiently abrogated Hrs expression in transduced cells (compare lane 1 with lanes 2–4 in Fig. 3a). Proliferation and survival were not affected by the shHrs knockdown (Hrs-KD) after 3 days of culture as compared to shCT cells (Fig. 3b). EEA1 and Rab5a immunolabeling revealed a strong enlargement of early endosome compartments upon Hrs-KD (Fig. 3c) as expected from previous works [35–37].

Differentiation of shCT and shHrs C2C12 myoblasts was evaluated after switch into Diff. After 96 h, myogenic differentiation was evaluated by MHC immunostaining. Hrs depletion strongly decreased myotube formation as shown by the low number of MHC-positive cells (Fig. 3d; compare panel d (1) with panel d (2–4)). Quantification of Hrs expression by Western blotting confirmed this result (compare lanes 1–6 with lanes 7–12 in panel e and quantifications in panel f). The negative impact of Hrs depletion on MHC expression correlated with a massive reduction of the myogenic index of Hrs-depleted cells (Fig. 3g).

Similar experiments were conducted in mouse primary myoblasts transduced with Hrs-shRNA#3 and shCT lentivectors. The data presented in Fig. 3h show efficient Hrs depletion (compare lanes 1 and 2) and a strong reduction of myotube formation after 2 days of differentiation as judged by the strong decrease of MHC-positive multinucleated fibers (see panel i) associated with a significant reduction of the myogenic index (panel j). Similar experiments were finally carried out on immortalized human myoblasts (Y711i cells) using three different human HRS-shRNAs. Our data revealed that HRS depletion in Y711i human cell line (Additional file 2 Fig. S2a) inhibited myotube formation (Additional file 2 Fig. S2b).

The specificity of the shHrs used in these experiments was confirmed by the rescue of shHrs#3-C2C12 differentiation by transfecting a non-targeted shRNA murine Myc-Hrs encoding construct or an empty vector as negative control. As shown in Additional file 2 Fig. S2c and S2d, the rescue of Hrs expression efficiently restored myotube formation (comparing the MHC marker in lane 3 with lane 4 in Additional file 2 Fig. S2c and the presence of MHC-positive myotubes in panel S2d).

To assess the specificity of Hrs requirement for myoblast differentiation, Tsg101-ESCRT-I, was depleted in C2C12 cells. Tsg101-shRNAs (Tsg101 #2 and #3) efficiently downregulated Tsg101 in C2C12 myoblasts (compare lane 1 with lanes 2–4 in Additional file 3 Fig. S3a). After 4 days in Diff, differentiated myotubes were observed in all conditions (Additional file 3 Fig. S3b). Tsg101 depletion (Tsg101#3-shRNA) impaired differentiation by 34% compared to the control condition as judged by the decrease of the myogenic index (see panel c). While Tsg101 depletion moderately affects the differentiation process, our data indicate that Hrs affect this process with more efficiency.

### The autophagy process is impaired in C2C12 Hrs-KD cells

The ESCRT machinery is tightly linked with the process of autophagy. Depletion of ESCRT components such as Hrs or Tsg101 was shown to compromise the autophagic degradation leading to the accumulation of the ubiquitinated binding proteins LC3 and SQSTM1/p62 (p62) in different cellular models [28–30, 38]. Interestingly, inhibition of autophagy precludes the differentiation and fusion of myoblasts [14]. During differentiation, the autophagic flux of C2C12 is enhanced and is characterized by a progressive accumulation of LC3-II and a decrease of p62. Conversely, blocking the autophagic flux significantly decreases LC3-II and prevents the differentiation of myoblasts into myotubes thus indicating that autophagy is required during myoblast differentiation [14]. When Hrs was depleted, strong accumulation of LC3-II and high sustained level of p62 was detected by Western blotting in C2C12 in Pro and Diff (compare lanes 1–6 with lanes 7–12 in Additional file 4 Fig. S4a and quantifications for p62 in panel S4b). This was confirmed by immunofluorescence confocal experiments using anti-LC3 and anti-p62 antibodies in Pro as well as at 4 days of differentiation (see white arrows in Additional file 4 Fig. S4c,d). Collectively these data reflect an impaired autophagic flux in absence of Hrs and in accordance with previous studies in other cellular models [29, 30].

Interestingly, Tsg101-KD cells led to similar results in immunoblotting (compare lanes 1–6 with lanes 7–12 in Additional File 4 Fig. S4e) as well as by immunofluorescence experiments (see white arrows in shTsg101-KD myoblasts in Pro and white arrowheads for myotubes in Diff in Additional file 4 Fig. S4f,g respectively).

Comparison of Hrs and Tsg101 depletion impacts on p62 accumulation in myoblasts revealed that Tsg101 KD induces a similar if not higher p62 accumulation compared to Hrs-KD (compare lane 2 with lane 3 in Additional file 5 Fig. S5a and quantifications in panel b).

Collectively, our results show that Hrs and Tsg101 are both required for proper autophagic flux in Pro and during differentiation suggesting that impaired autophagy partly contributes to the defect in C2C12 differentiation upon both Hrs or Tsg101 depletion.

### Hrs depletion strongly affects the function of myogenic regulatory factors

**Fig. 4:**
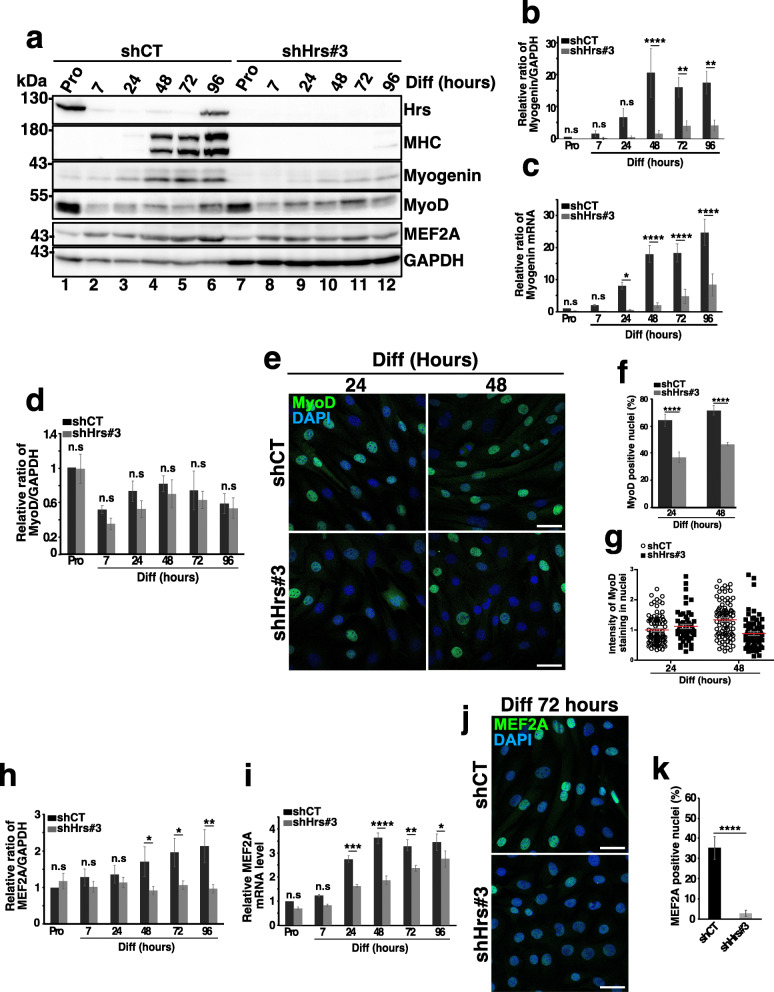
Depletion of Hrs affects the expression and localization of the myogenic regulator factors. **a** Representative Western blotting of shCT and shHrs#3 C2C12 extracts in Pro status (lanes 1 and 7) and at 7, 24, 48, 72, and 96 h of differentiation (lanes 2–6 and 8–12). Membranes were probed with the anti-HRS, -MHC, -Myogenin, -MyoD, and -MEF2A antibodies. GAPDH was used as a loading control. **b** Quantifications of the myogenin protein level present in experiments like in **a**. Data are presented as the ratio of myogenin/GAPDH and normalized to the Pro starting point condition. Data represent mean +/− SEM, *n* = 4 experiments. Significance was assessed using a two-way ANOVA test; **p < 0.01, ****p < 0.0001. ns not significant. **c** qRT-PCR of myogenin mRNA realized on mRNAs from shCT and shHrs#3 C2C12 cells collected in Pro and at 7, 24, 48, 72, and 96 h of differentiation. Data are normalized to the Pro starting point condition and presented as mean +/− SEM, *n* = 4 experiments. Significance was assessed using a two-way ANOVA test; *p < 0.05, ****p < 0.0001. ns not significant. **d** Quantification of the MyoD protein level present in experiments like in **a**. Data are presented as the ratio of MyoD/GAPDH signals and normalized to the Pro starting point condition. Data represent mean +/− SEM, *n* = 5 experiments. Significance was assessed using a two-way ANOVA test. ns not significant. **e** Representative immunofluorescence images of MyoD in shCT and shHrs#3 C2C12 cells at 24 or 48 h of differentiation and probed with anti-MyoD (green) and DAPI (blue). Scale bar, 40 μm. **f** Quantification of high MyoD-positive nuclei. The data represent mean +/− SEM, corresponding to 9 fields counted per experiment, *n* = 3 experiments. Significance was assessed using a Mann–Whitney *U* test; ****p < 0.0001. **g** Representative quantification of MyoD staining in nuclei. The mean intensity of each positive MyoD nuclei has been measured in each condition. In total, 55–92 nuclei have been counted in each condition. **h** Quantification of the MEF2A protein level present in experiments like in **a**. The data are presented as the ratio of MEF2A/GAPDH signals and normalized to the Pro starting point condition. Data represent mean +/− SEM, *n* = 5 experiments. Significance was assessed using a two-way ANOVA test; *p < 0.05, **p < 0.01. ns not significant. **i** qRT-PCR of mef2A mRNA from shCT and shHRS#3 C2C12 cells collected in Pro and at 7, 24, 48, 72, and 96 h of differentiation. The data represent mean +/− SEM, *n* = 4 experiments. Significance was assessed using a two-way ANOVA test; *p < 0.05; **p < 0.01; ***p < 0.001; ****p < 0.0001. ns not significant. **j** Representative immunofluorescence images of MEF2A distribution (green) in shCT and shHrs#3 C2C12 cells and analyzed at 72 h of differentiation. DAPI staining (blue). Scale bar, 40 μm. **k** Quantification of high MEF2A-positive nuclei. Data represent mean +/− SEM, corresponding to 7 fields counted per experiment, *n* = 3 experiments. Significance was assessed using a Mann–Whitney *U* test, ****p < 0.0001

Myogenin expression is indispensable for the late stages of myoblast differentiation, i.e., myoblast fusion and MHC expression [39]. In shCT cells, myogenin expression significantly increased from 24 to 96 h of culture in Diff (see Fig. 4a lanes 1–6 and quantifications in panel b). In Hrs-depleted cells, myogenin upregulation was strongly reduced (compare lanes 1–6 with lanes 7–12 and quantifications in panel b) both at the protein and mRNA levels (Fig. 4a–c), thus indicating that Hrs depletion impairs the myogenin expression induction.

The myogenin induction requires both MyoD and MEF2 transcription factors [3, 39, 40]. This prompted us to investigate the expression level of MyoD in shCT and shHrs cells in Pro or during the differentiation. MyoD expression was not significantly affected by Hrs depletion (comparing lanes 1–6 with lanes 7–12 in Fig. 4a and quantifications in panel d). Interestingly, confocal immunofluorescence microscopy analyses revealed a decrease of MyoD-positive nuclei in shHrs cells at 24 and 48 h of differentiation compared to shCT cells (see panel e and quantifications in panel f) as well as a decrease of MyoD level intensity at 48 h (see panel g). These observations suggest that alteration of MyoD nuclear translocation could explain the reduction in myogenin upregulation in Hrs-KD cells.

We next analyzed the MEF2A protein and mRNA levels in shCT and shHrs cells. While MEF2A level was upregulated from 48 to 96 h of differentiation in shCT, it remained at its basal level in shHrs cells (see Fig. 4h). qRT-PCR revealed that increase in mef2A mRNA was significantly delayed in shHrs compared to shCT cells (Fig. 4i). Consequently, MEF2A nuclear levels were significantly reduced in shHrs cells as shown in Fig. 4j,k. Collectively, our data indicate that Hrs-KD significantly inhibits the expression and/or the nuclear accumulation of essential myogenic regulators.

### Depletion of Hrs, but not of Tsg101, inhibits myogenesis via MEK/ERK activation

Hrs regulates the trafficking and the degradation of cell surface receptors including RTKs that control downstream signaling pathways such as the Ras-Raf-MEK1/2-ERK1/2 pathway [22–24, 41]. Interestingly, constitutively active mutants of N-Ras and H-Ras block differentiation in C2C12 mouse myoblasts [42, 43] and activation of the Ras/Raf/MEK/ERK pathway was also found to impair MEF2A nuclear translocation, myogenin upregulation, and thus myoblast differentiation [43, 44]. These observations prompted us to investigate the impact of Hrs depletion on the MEK/ERK signaling pathway.

**Fig. 5:**
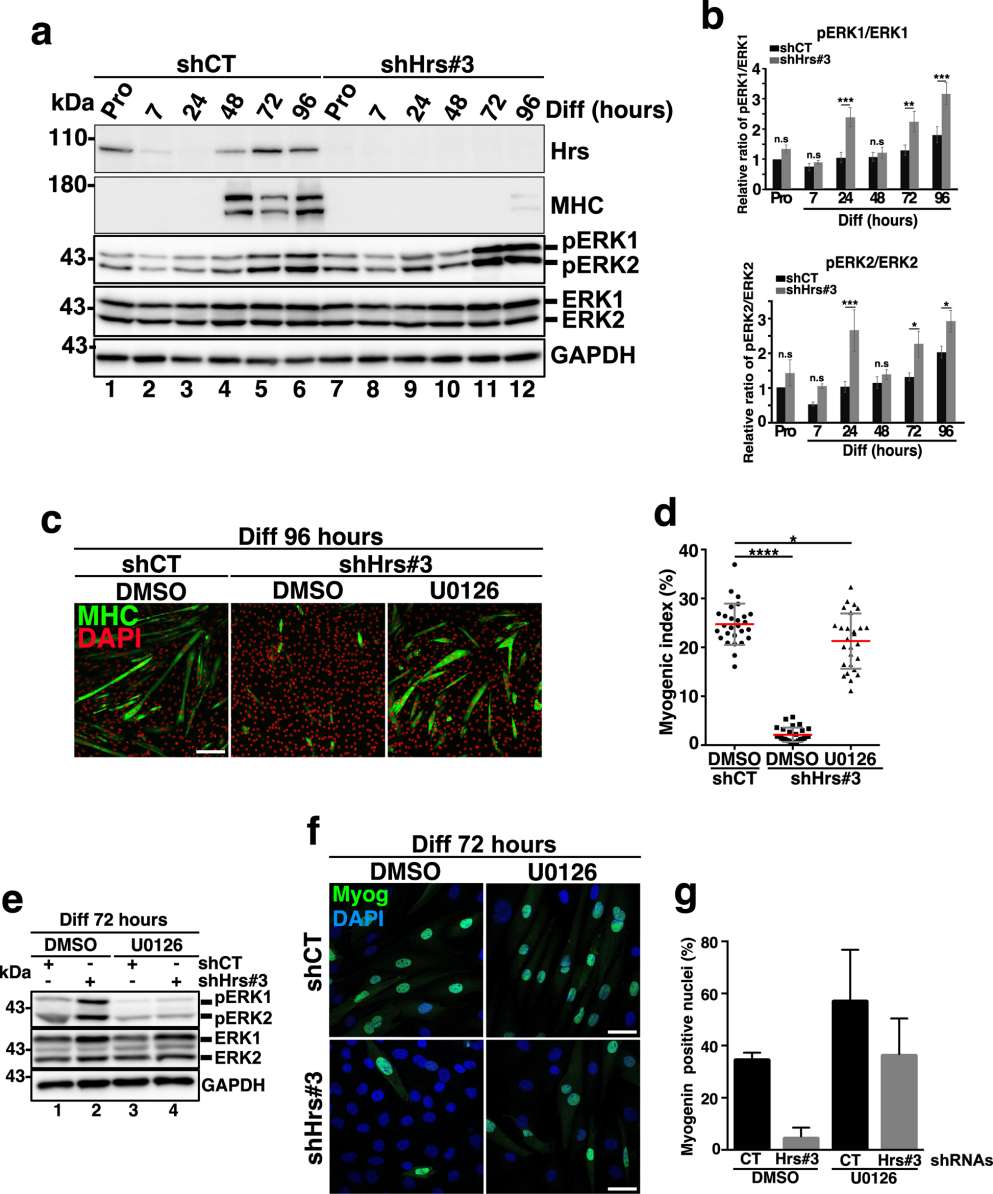
The MEK1/2/ERK1/2 pathway is upregulated into Hrs-depleted cells and its inhibition rescues formation of myotubes. **a** Representative Western blotting of protein extracts from shCT and shHrs#3 C2C12 collected in Pro (lanes 1 and 7) and at 7, 24, 48, 72, and 96 h of differentiation (lanes 2–6 and 8–12) and probed with anti-HRS, -MHC, -pT202/Y204-ERK1/2, and -total-ERK1/2 antibodies. GAPDH was used as a loading control. **b** Quantification of the phosphorylated-ERK1/2 from similar experiments presented in **a**. The data are presented as a ratio of pERK1/ERK1 and pERK2/ERK2 and normalized to the Pro starting point condition. Data represent mean +/− SEM, *n* = 3 experiments. Significance was assessed using a two-way ANOVA test; *p < 0.05; **p < 0.01; ***p < 0.001. ns not significant. **c** Representative immunofluorescence images of shCT and shHrs#3 transduced C2C12 at 96 h of differentiation upon MEK1/2 inhibitor U0126 (10 μM) or DMSO treatment and stained with anti-MHC antibody as a marker of myotubes (green) and DAPI (red). Scale bar, 200 μm. **d** Quantification of the myogenic index corresponding to the number of nuclei present in MHC-positive structures in experiments as presented in **c**. Data are mean +/− SEM, each dot represents one field, *n* = 27 images (*n* = 3 experiments). Significance was assessed using a Mann–Whitney *U* test; *p < 0.05; ****p < 0.0001; ns not significant. **e** Representative Western blotting of shCT and shHrs#3 cell extracts collected at 3 days of differentiation in presence of DMSO or 10 μM of U0126 and probed with anti-pT202/Y204-ERK1/2 and -total-ERK1/2 antibodies. GAPDH was used as a loading control. **f** Representative immunofluorescence images of shCT and shHrs#3 transduced C2C12 at 72 h of differentiation upon treatment with either 10 μM of the U0126 MEK1/2 inhibitor or DMSO and stained with the anti-myogenin (red) and DAPI (blue) for nuclear staining. Scale bar, 40 μm. **g** Quantification of the myogenin-positive nuclei (%). Data represent mean +/− SEM, 10 fields have been counted per experiments, *n* = 2 independent experiments

For this purpose, we first evaluated by Western blotting the extracellular regulated kinase 1 and 2 (ERK 1/2) levels and phosphorylation status during the course of differentiation. ERK1/2 global levels did not vary significantly between shCT and shHrs#3 cells (compare lanes 1–6 to lanes 7–12 in Fig. 5a). Conversely, ERK1/2 phosphorylation (pERK1/2) was significantly increased at 24 h in shHrs#3 cells compared to shCT (compare lane 3 with lane 9 and lanes 5–6 to lanes 11–12 in panel a and quantifications in panel b), indicating an upregulation of this pathway in the early steps of differentiation in Hrs-KD.

To investigate if activation of the ERK/MEK pathway was responsible for impaired myogenesis in Hrs-depleted cells, shCT and shHrs cells were cultured in presence or absence of the U0126 MEK/ERK inhibitor [43, 45] for 96 h in Diff. Treatment with the U0126 inhibitor efficiently rescued the differentiation of Hrs-KD cells as judged by the significant restoration of MHC labeling and myotube formation in shHrs-treated cells (Fig. 5c and the myogenic index in panel d). Analysis by Western blotting of U0126-treated and untreated shCT and shHrs#3 cells at day 3 of differentiation confirmed the efficiency of the compound since ERK1/2 phosphorylation was significantly decreased (compare lane 1 with lane 3 and lane 2 with lane 4 in panel e). Immunofluorescence experiments confirmed that myogenin expression was significantly restored in Hrs-depleted cells at day 3 of differentiation upon U0126 addition (Fig. 5f,g). Collectively, these data show that MEK/ERK inhibition rescues myoblasts differentiation in the absence of Hrs. On the opposite, ERK1/2 and pERK1/2 levels were similar between shTsg101 and shCT at the early steps of differentiation (Additional file 6 Fig. S6a and quantifications in panel b) thus indicating that Tsg101 alters myogenesis independently of this signaling pathway.

### Hrs depletion impairs the degradation of the EGFR receptor tyrosine kinase RTK in C2C12

The RTK family regulates several signaling pathways that may act positively or negatively on the process of myogenic differentiation. For instance, both EGFR and FGFR are negative regulator of myogenesis that need to be downregulated during differentiation [46, 47]. Importantly, Hrs has a general role in regulating the trafficking, sorting, and degradation of diverse classes of signaling receptors including RTKs thus negatively modulating their activities [22, 24, 41, 48]. For example, Hrs depletion in the HeLa cellular model was previously shown to compromise the degradation of the EGFR, one of the best characterized RTK member, resulting in its accumulation into the early endosomal compartments and in the activation of the downstream MEK/ERK1/2 signaling pathway [24, 49].

**Fig. 6:**
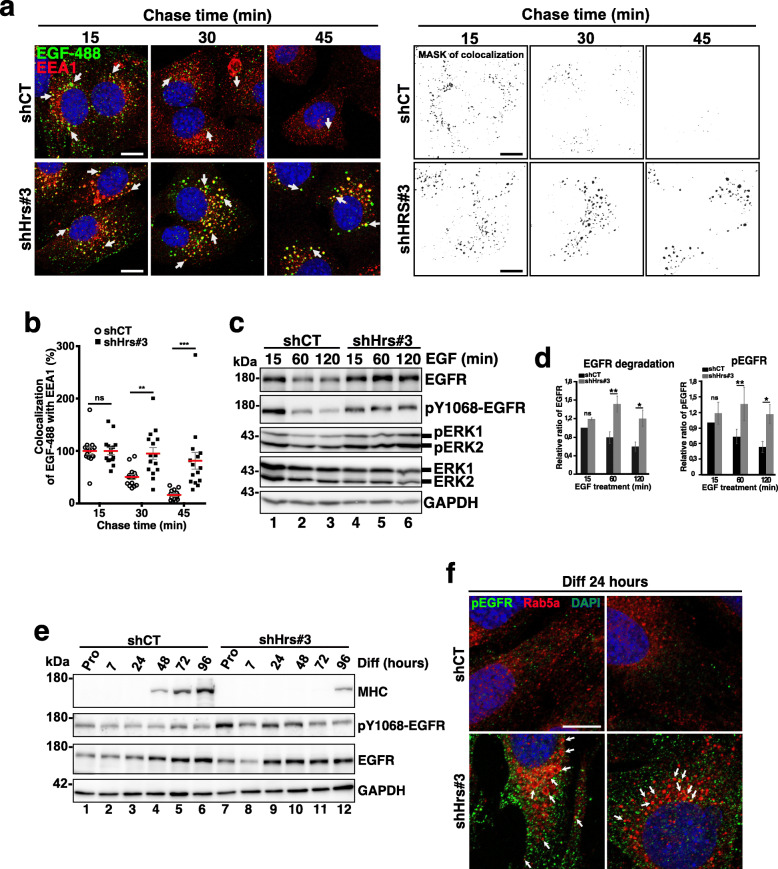
EGFR is accumulated in Hrs-depleted cells and its activation partially correlates with induction of the MEK1/2/ERK1/2-pathway. **a** Left panel: representative immunofluorescence images of pulse-chase experiments: shCT and shHrs#3 C2C12 were stimulated for 5 min with 50 ng/mL of EGF-488 (green) and after removing unbound ligand-chased for the indicated amount of time. Cells were probed with the anti-EEA1 to visualize EE and trafficking of EGF-488/EGFR was followed through the EE pathway. White arrows indicate colocalization of EGF-488 (in green) with the EEA1 EE marker (red) and DAPI (blue). Note the accumulation of EGF-488 in EEA1-positive compartments (yellow merge) in shHrs#3-depleted cells. Right panel: mask of colocalization between EGF-488- and EEA1-positive compartments. Scale bars, 20 μm. **b** Quantifications of colocalization of EGF-488 in EEA1 compartments. Analysis shows the percentage of EGF-488 overlapping with EEA1 and establishes the endocytic trafficking of EGF ligand through the EE pathway (EEA1 compartment). Open circles correspond to shCT and black squares to shHrs#3 conditions. The data represent mean +/− SEM. Analyses were done on 5 fields per condition on *n* = 3 experiments. Significance was assessed using a Mann–Whitney *U* test; ***p < 0.001; **p < 0.005. ns not significant. **c** Degradation of EGFR. shCT and shHrs#3 cells were stimulated with 50 ng/mL of EGF and with 10 μg/mL of cycloheximide to inhibit de novo synthesis of EGFR. Cells were recovered 15 (lanes 1,4), 60 (lanes 2,5), and 120 (lanes 3,6) min after stimulation and cells extracts were analyzed by Western blotting using anti-EGFR, -pY1068-EGFR, -ERK1/2, and -pT202/Y204ERK1/2. GAPDH was used as a loading control. **d** Quantifications of EGFR (left panel) and pY1068-EGFR (right panel) protein levels. Data are presented as the ratio of EGFR/GAPDH and pY1068-EGFR/GAPDH. Data are mean +/− SEM, *n* = 3–4 experiments. Significance was assessed using a two-way ANOVA test; **p < 0.01, ****p < 0.0001; ns not significant. **e** Representative Western blotting of protein extracts from shCT and shHrs#3 C2C12 collected in Pro (lanes 1 and 7) and at 7, 24, 48, 72, and 96 h of differentiation (lanes 2–6 and 8–12) and probed with anti-MHC, -EGFR, -pY1068-EGFR, and -GAPDH. **f** Representative immunofluorescence images of shCT and shHrs#3 C2C12 at 24 h of differentiation and probed with anti-pY1068-EGFR (green), -Rab5a (red) for EE and DAPI (blue). White arrows showed the pY1068-EGFR signal associated with Rab5a vesicles. Scale bar, 10 μm

Collectively, these observations prompted us to investigate a potential contribution of RTK deregulation in the deficient differentiation phenotype observed in Hrs-depleted cells. To this aim, we have focused our attention on EGFR and analyzed its cellular trafficking upon Hrs depletion in our C2C12 cellular model. Pulse-chase experiments performed with Alexa-488-tagged EGF allowed to follow the colocalization of liganded EGFR with the EEA1 early endosomal marker in shCT and shHrs#3 KD cells. In shCT cells, the EGF-488 signal strongly decreased after 30 to 45 min of chase certainly due to its final trafficking to the degradative compartments (Fig. 6a, b [49];). Conversely, in shHrs cells, the EGF-488 signal progressively accumulated in EEA1-positive early endosomes (see the merged-yellow punctuated EGF-488/Red-EEA1 co-occurrence signals in left panel of Fig. 6a and the associated mask of colocalization in the right panel). Quantifications corresponding to the percentage of co-occurrences measured in experiments as presented in panel a confirmed that Hrs depletion in myoblast alters the trafficking of EGF-488 and induces its accumulation in EEA1-positive compartments (see Fig. 6b). To determine if EGF-488 accumulation in EEA1-positive compartments was specific to Hrs loss of function, similar experiments were carried out in Tsg101-KD cells. Tsg101 depletion did not alter EGFR trafficking in C2C12 since no accumulation of EGF-488 was observed in EEA1 compartments (Additional file 6 Fig. S6c,d; see “Discussion”).

To determine if Hrs depletion impairs the degradation of EGFR, shCT and shHrs#3 myoblasts were cultured in presence of EGF stimulation and cycloheximide. EFGR protein level was assessed by Western blotting at 15, 60, and 120 min after stimulation. While a progressive decrease of EGFR was observed in shCT cells, EGFR protein level was maintained during this period in shHrs#3 cell (compare lanes 1–3 with lanes 4–6 in panel c and quantifications in panel d, left panel) thus indicating that EGFR degradation is impaired compared to shCT cells and correlating with previous published data [49]. Analyses of the activated form of EGFR that is phosphorylated on Tyr1068 (Y1068-EGFR) [50] in the same conditions revealed that its accumulation was associated with the reduced degradation of EGFR (see right panel d,) as well as the upregulation of pERK1/2 (panel c). Collectively, these data confirm that Hrs depletion alters the trafficking and the degradation process of this RTK. On the opposite, EGFR degradation was not impaired in Tsg101-depleted cells and follows the same kinetic as observed in shCT cells (see Additional file 6 Fig. S6e,f), correlating with the absence of accumulation of EGF-488 pulse-chase experiments realized on Tsg101-depleted cells (Additional file 6 Fig. S6c,d) and confirming the difference between Tsg101 and Hrs depletion impact on the EGFR trafficking in the C2C12 cellular model (see “Discussion”).

Immunoblotting analyses of pY1068-EGFR in shHrs cells revealed an upregulation during the early steps of differentiation (Fig. 6e). This observation was further confirmed by confocal immunofluorescence imaging at 24 h of differentiation showing that pY1068-EGFR signal was mainly distributed in the cytoplasm and associated with Rab5a-positive early endosomes (see white arrows) in Fig. 6f. Conversely, only a very faint signal was observed in shCT cells in the same conditions.

We next asked if the moderate effect of Tsg101 depletion on the differentiation process could be explained by the recruitment of ESCRT-III complex through the Ptpn23/HD-PTP Bro1-containing accessory protein independently of Tsg101-ESCRT-I. To investigate the role of this parallel arm, we focused our attention on Ptpn23/HD-PTP protein that was previously involved in the sorting and degradation of EGFR [51, 52]. C2C12 cells were transduced with lentivector encoding shRNAs depleting Ptpn23/HD-PTP versus shCT. The most efficient Ptpn23/HD-PTP shRNA (shRNA#2; compare lane 1 with lane 3 in additional file 7 Fig. S7a) strongly impaired the differentiation of myoblasts into myotubes (see panels S7b,c) reminding our previous observations when Hrs was depleted. Interestingly, Ali et al. [51] showed that depletion of HD-PTP in HeLa cells was associated with a strong reduction in the protein level of HRS. We thus analyzed the protein level of Hrs in Ptpn23/HD-PTP-depleted C2C12 cells and found that Hrs protein level was also strongly reduced in this model (compare lane 1 with lane 3 for Ptpn23/HD-PTP signal). These results demonstrate that the parallel arm mediated by Ptpn23/HD-PTP also controls differentiation via Hrs, highlighting the central role of Hrs in this process.

### Akt2/FOXO1 signaling is impaired in muscle cells lacking Hrs but not Tsg101

**Fig. 7:**
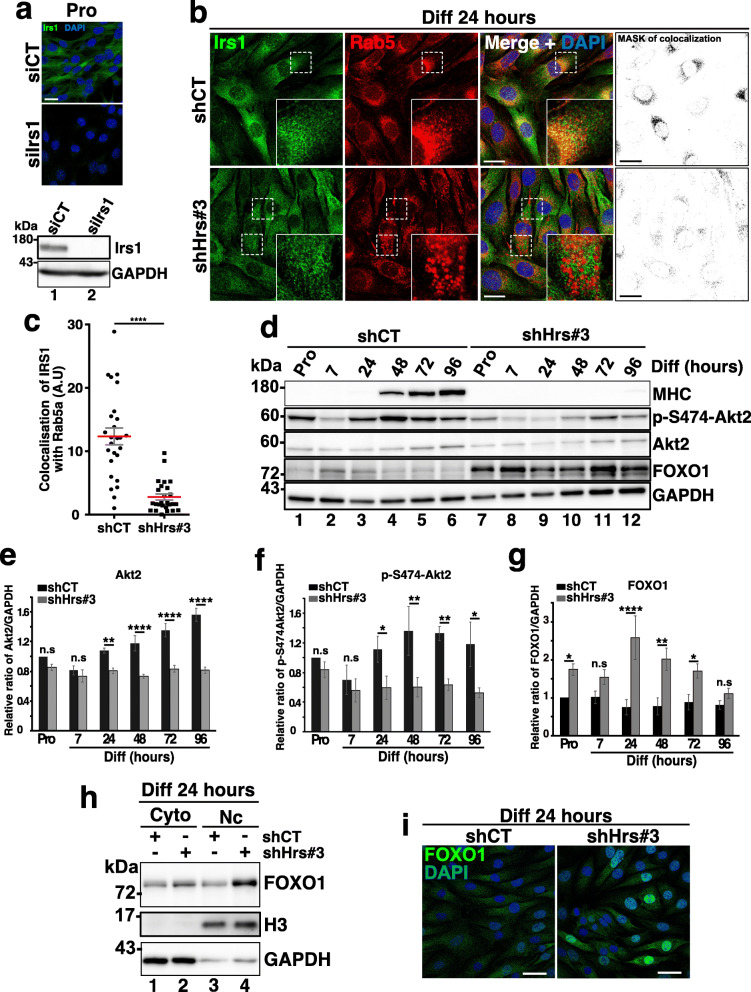
Depletion of Hrs downregulates Akt2 signaling and activates FOXO1. **a** Upper panel: specificity control of Irs1 antibody. Representative immunofluorescence images of siCT and siIrs1 in C2C12, probed with anti-Irs-1 (green) and DAPI (blue). Scale bars, 20 μm. Lower panel: Western blotting of siCT and siIrs1 C2C12 probed with anti-Irs1. GAPDH was used as a loading control. **b** Left panel: representative immunofluorescence images of shCT and shHrs#3 C2C12 at 24 h of differentiation and probed with anti-Irs1 (green), -Rab5a (red) for EE and DAPI (blue). White dotted squares represent a focus of the selected regions (see insets). The right panel represents the mask of colocalization between Irs1 and Rab5a. Scale bars, 20 μm. **c** Quantifications of colocalized Irs1/Rab5a as presented in **a** have been made by creating binary mask between merge channel of Irs1 and Rab5a. Quantification of the signals obtained is presented where one dot corresponds to one image. Data represent mean +/− SEM, 8 images for each group, *n* = 3 experiments. Significance was assessed using a Mann–Whitney *U* test, ***P = 0.001. **d** Representative Western blotting of shCT and shHRS#3 C2C12 extracts collected in Pro (lanes 1 and 7) and at 7, 24, 48, 72, and 96 h (lanes 2–6 and 8–12) of differentiation and probed with anti-MHC, -p-S474-Akt2, -Akt2, and -FOXO1. GAPDH was used as a loading control. **e** Quantification of total Akt2 protein level in experiments as presented in **c**. Data are presented as ratio of Akt2/GAPDH signal and normalized to the starting point Pro condition. Data represent mean +/− SEM, *n* = 3 experiments. Significance was assessed using a two-way ANOVA test; ***p < 0.001; ****p < 0.0001; ns not significant. **f** Quantification of p-S474-Akt2 protein level in experiments as presented in **c**. Data are presented as ratio of pS474-Akt2/GAPDH signal and normalized to the starting point Pro condition. Data represent mean +/− SEM, *n* = 3 experiments. Significance was assessed using a two-way ANOVA test; *p < 0.05; **p < 0.01; ns not significant. **g** Quantification of total FOXO1 protein level in experiments as presented in **c**. Data are presented as ratio of FOXO1/GAPDH signal and normalized to the starting point Pro condition. Data represent mean +/− SEM, *n* = 3 experiments. Significance was assessed using a two-way ANOVA test; *p < 0.05; **p < 0.01; ****p < 0.0001; ns not significant. **h** Representative Western blotting of nuclear and cytosolic fractionation of shCT and shHrs#3 transduced C2C12 at 24 h of differentiation and probed with the anti-FOXO1, -H3 (for nuclear marker). GAPDH was used as a loading control. **i** Representative immunofluorescence images of shCT and shHrs#3 C2C12 at 24 h of differentiation and probed with anti-FOXO1 (green) and DAPI (blue). Scale bars, 40 μm

The insulin receptor substrate 1 (Irs1) mediates Akt activation by insulin growth factor receptor (IGFR) upon IGF binding. IGFR/Irs1/Akt2 signaling is indispensable for the myoblast differentiation [53]. Cong et al. [10] recently showed that Rab5a interacts with Irs1 and activates Irs1 by promoting Irs1-IGFR localization on early endosomes thereby upregulating Akt signaling [10]. Since Hrs depletion induced the formation of enlarged Rab5a-positive early endosomes (see Fig. 3c), Irs1 trafficking and distribution were investigated by immunofluorescence in Hrs-KD cells. Specificity of Irs1 antibody was first validated by immunofluorescence experiments and Western blotting analyses in Irs1-depleted C2C12 myoblasts (see Fig. 7a upper and lower panels respectively). In line with the observations of Cong et al. [10], we found that Irs1 partially colocalized with Rab5a in shCT cells (see panel b). However, despite an accumulation of enlarged Rab5a-positive early endosomes in Hrs-depleted cells, we failed to observe an increased recruitment and enrichment of Irs1 in these compartments (see Fig. 7b,c). Collectively, these data suggest that altered recruitment of Irs1 by Rab5a in early endosomal compartments could be associated to impaired Irs1 downstream signaling in Hrs-depleted cells. To verify this hypothesis, we investigated Akt2 signaling which has been shown to be regulated by Irs1 in C2C12 [53]. The total Akt2 level and its phosphorylation on Ser474, an indicator for its activity, were both significantly decreased in absence of Hrs compared to control (compare lanes 1–6 to lanes 7–12 in Fig. 7d and quantifications in panels e and f) thus demonstrating downregulation of Akt2 signaling in Hrs-KD cells.

Nuclear exclusion of the FOXO1 transcription factor has been shown to be required for myogenic differentiation while Akt inhibition induces the upregulation of FOXO1 expression and nuclear translocation (reviewed in [54]). Consistently with impaired Akt2 signaling, we found that FOXO1 level was upregulated in absence of Hrs (compare lanes 1–6 with lanes 7–12 in Fig. 7d and quantifications in panel g). Cytosol/nucleus fractionations were then realized on shCT and shHrs cells to analyze the distribution of FOXO1 at the early phase of differentiation. After 24 h of differentiation, high nuclear FOXO1 content was observed in shHrs compared to shCT cells (compare lanes 3 and 4 in Fig. 7h and FOXO1 signal in immunofluorescence experiment in Fig. 7i). Conversely, no upregulation of FOXO1 protein level was observed in Tsg101-depleted cells (Additional file 8 Fig. S8). Collectively, our data indicate that absence of Hrs, but not of Tsg101, may impair myogenesis via the alteration of the Akt2/FOXO1 signaling pathway.

### Gsk3β is another potential factor involved in the Hrs-KD-differentiation defect phenotype

**Fig. 8:**
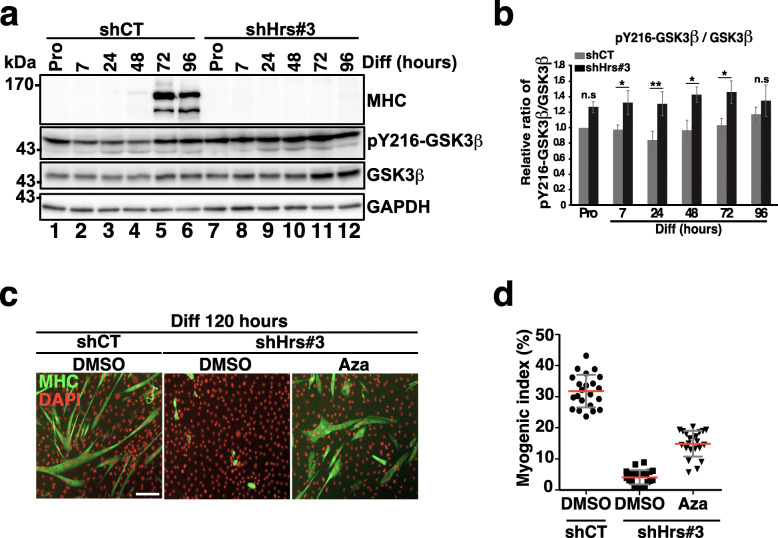
Inhibition of GSK3β partially rescues myoblast differentiation. **a** Representative Western blotting of shCT and shHrs#3 C2C12 cell extracts collected in Pro (lanes 1 and 7) and at 7, 24, 48, 72, and 96 h (lanes 2–6 and 8–12) of differentiation and probed with the anti-MHC, -GSK3β, and -phosphorylated-Tyr216 (pY216-GSK3β). GAPDH was used as a loading control. **b** Quantification of the pY216-GSK3β level in experiments as in **a**. Data are presented as ratio of pY216-GSK3β/total GSK3β and normalized to the starting point Pro condition as mean +/− SEM, *n* = 4 experiments. Significance was assessed using a two-way ANOVA test; *p < 0.05; **p < 0,01; ns not significant. **c** Representative immunofluorescence images of shCT and shHrs#3 C2C12 transduced cells treated with 3 μM of azakenpaullone (Aza) GSK3β-inhibitor or DMSO as negative control and analyzed at 120 h of differentiation. Cells were stained with anti-MHC (green) and DAPI (red). Scale bar, 200 μm. **d** Quantification of the myogenic index (differentiation index) corresponding to the number of nuclei present in MHC-positive structures as depicted in **c**. Data are presented as mean +/− SEM with 10 images per condition. *n* = 2 experiments

The glycogen synthase kinase 3 beta (GSK3β) is a serine-threonine kinase that is involved downstream of multiple cellular signaling pathways [55]. The active form of GSK3β that is phosphorylated on Tyr216 (Y216-GSK3β [56]) was shown to act as a negative regulator of skeletal muscle differentiation [57] while inhibition of GSK3β has been shown to enhance myotube formation [58]. Interestingly, Taelman et al. observed that GSK3β accumulates in the cytoplasm of HRS-depleted HeLa cells [26]. This prompted us to investigate the impact of Hrs depletion on GSK3β expression and activity in muscle cells. For this purpose, we first analyzed the expression level of total GSK3β and of its active phosphorylated isoform pY216-GSK3β by Western blotting. The data presented in Fig. 8a,b indicated that the level of activated pY216-GSK3β was significantly increased in Hrs-depleted cells from the early steps of differentiation up to 72 h (comparing lanes 1–6 with lanes 7–12 in panel a and quantifications in panel b) indicating increased GSK3β signaling. To determine if activation of GSK3β linked to Hrs depletion could participate to the differentiation defect phenotype, we tested whether the azakenpaullone (Aza)-specific GSK3β inhibitor could rescue the differentiation of Hrs-depleted cells. To this aim, shCT and shHrs#3 cells were cultured in differentiation medium in absence (DMSO) or presence of Aza (3 μM) for 5 days. At day 5 of differentiation, cells were recovered and analyzed by immunofluorescence microscopy using the anti-MHC. Our data revealed that Aza treatment restores the formation of myotube fibers in shHrs#3 KD cells as judged by the MHC labeling (see the restauration MHC labeling Fig. 8c) and the myogenic differentiation index (see panel d) whereas non-treated Hrs-KD remained undifferentiated. Overall, these results demonstrate that GSK3β is an additional myogenic signaling factor controlled by Hrs which contribute to the differentiation defects in Hrs-KD cells.

### Hrs is upregulated in dystrophic mouse models

To determine if Hrs expression is modulated in regenerating muscles, we performed Western blotting analyses on tibialis anterior muscle extracts from two well characterized dystrophic mouse models Duchenne muscular dystrophy (mdx model [59];) and the muscle-specific mTOR knockout (mTORmKO model [60];). Interestingly, we observed a significant upregulation of Hrs protein level in both mdx (compare lanes 1–4 with lanes 5–8 in Additional file 9 Fig. S9a and quantifications in panel S9b) and mTORmKO (comparing lanes 1–4 with lanes 5–8 in Additional file 9 Fig. S9c and quantifications in panel S9d) muscle samples.

Collectively, these data show the tight relationships between Hrs expression and the pathophysiological status of muscle tissue.

## Discussion

**Fig. 9:**
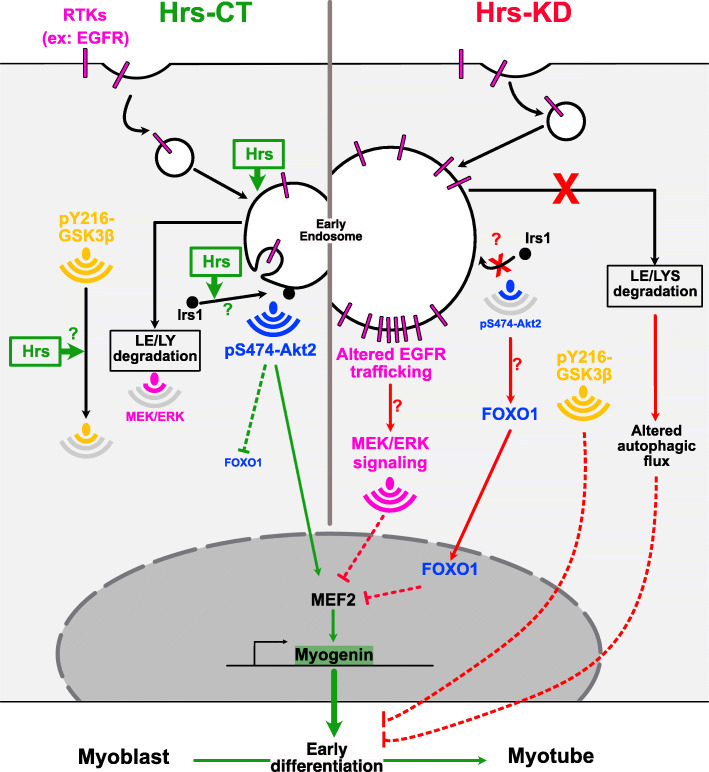
Schematic summary of the findings. We found that Hrs depletion strongly inhibits myogenesis. Mechanistically, Hrs silencing impairs MRFs in the early steps of differentiation through the upregulation of the MEK/ERK signaling pathway concomitant with impaired Akt2 signaling network and activation of the downstream FOXO1 repressor of myogenesis. Upstream, degradation of EGFR, a regulator of the MEK/ERK signaling, was impaired in absence of Hrs. The activity of the myogenic repressors GSK3β was also found to be upregulated when Hrs was absent. Inhibition of GSK3β by the azakenpaullone GSK3β-specific inhibitor compound significantly restores the impaired differentiation observed in Hrs-depleted cells. In addition, functional autophagy that is required for myogenesis was also found to be strongly inhibited. Left panel: under normal condition. Right panel: upon Hrs depletion

In this study, we investigated the role of Hrs-ESCRT-0 in muscle cell differentiation and showed that Hrs is crucially required at the onset of myogenesis by controlling cellular trafficking, signaling, and degradation pathways (resumed in Fig. 9).

Hrs is transiently degraded by the proteasome at the onset of differentiation when myoblasts are switched from Pro to Diff. This is reminiscent of Hrs degradation induced by starvation [31, 32].

The myogenic process is accompanied by a strong reorganization of intracellular compartments from disperse cytoplasmic distribution during proliferation to ring perinuclear shape in differentiated myotubes [11, 61, 62]. Here we observed that Hrs localization and distribution follows the dynamic of this trafficking during differentiation. We found that Hrs colocalized with Rab5a and CHC in this perinuclear ring region. The molecular and cellular mechanisms involved in the strong reorganization of cellular compartments during differentiation are still non-elucidated. At the present time, we do not know if Hrs could play a role in the dynamic restructuration and relocalization of the endosomal network to organize functional contractile myotubes. Several studies opened new insights on a possible role of Hrs in such reorganization. For example, Hrs depletion was found to strongly impair the recruitment of CHC at the limiting membrane of early endosomes in clathrin bilayer/flat clathrin coat microdomains in HeLa cells [33, 63, 64] while CHC depletion in muscle cells disrupts the α-actinin striated pattern leading to a strong disorganization of actin filaments in myotubes [12]. Hrs was also required to localize the Actin nucleating factor WASH to the endosomal compartments or to bind Actinin-4, both important for an efficient recycling of membrane receptors [20, 21]. Interestingly, silencing of Actinin-4 impaired C2C12 differentiation [65]. More recently, protein level of Kif13A, a kinesin involved in the morphogenesis and positioning of tubular recycling endosomes [66], was strongly reduced in Hrs-depleted models [67]. Altogether, it is thus tempting to speculate that Hrs might participate to this reorganization directly or indirectly through the recruitment of binding partners like CHC or by modulating cellular factors involved in the cytoskeleton dynamic and/or the positioning of compartments in the differentiated myotube structures.

Our functional analysis revealed that Hrs depletion strongly impaired myoblasts differentiation, whereas loss of Tsg101 only partially inhibited this process. Interestingly, during the course of our study, Hunt et al. reported that Hrs depletion impaired the differentiation of myoblasts into myotubes in the drosophila and mouse models [68]. Conversely, silencing of the Hrs binding partner ESCRT-0 Stam component had no effect on the differentiation [68].

It is worth noticing that while Hrs, Stam, or Tsg101 are affiliated to the same ESCRT machinery and act in a sequential way during MVB biogenesis, the impact of Hrs-KD on the C2C12 differentiation is more deleterious than that of the other ESCRT components tested so far. Interestingly, our investigations also revealed that silencing of the Ptpn23/HD-PTP ESCRT accessory protein strongly affects the process of differentiation. However, we also observed a strong concomitant reduction of Hrs protein level in absence of Ptpn23-HD-PTP correlating with previous published data in HeLa cells [51]. Collectively, this data suggest that Hrs is a keystone regulating important factors involved in myogenesis.

While the molecular and cellular mechanisms underlying Hrs-mediated myogenesis regulation were not investigated by Hunt et al., we identified several potential Hrs-regulated processes that may be crucial for myoblast differentiation.

Skeletal muscle differentiation is initiated by the coordinated action of master transcription factors, including MyoD, MEF2, and myogenin. MyoD and MEF2 transcription factors activate the expression of myogenin, which then cooperate with MEF2 transcription factors to drive terminal differentiation [3, 4, 44, 69]. Altogether, we found that both MyoD and MEF2A ability to activate transcription was compromised by Hrs depletion, thus explaining the lack of activation of myogenin expression and the consecutive defect of myoblasts fusion and expression of the late differentiation marker MHC.

The role of the MEK/ERK signaling pathway in myogenesis is versatile and time window dependent. In the early phase, MEK and ERK activation repress myogenesis [2, 69–72] whereas inhibition of ERK2 in later steps prevents fusion of myoblasts into multinucleated myotubes [71]. Our investigations revealed that Hrs silencing is associated with an upregulation of MEK/ERK signaling. We found that myogenin expression and differentiation were restored by the MEK/ERK inhibitor U0126 demonstrating that upregulation of MEK/ERK signaling is a cause for myogenesis inhibition. In the same time, our data revealed that Hrs depletion impairs the degradation of EGFR, a well-known regulator of the MEK/ERK pathway, which is further upregulated. These data are consistent with those previously published indicating that EGFR downregulation is an early event required for the induction of myoblast differentiation allowing the MEF2A and myogenin induction [46]. Moreover, Raf constitutive activation was also shown to impair murine L6 myoblasts differentiation by altering MEF2A trafficking [44] and MEK activation was shown to repress the ability of MyoD to transactivate its target genes [69].

While we did not observe modification of MyoD expression, MyoD translocation into the nucleus was decreased in Hrs-KD cells. At this stage, the mechanisms by which Hrs depletion affects the trafficking of MyoD remains unresolved.

Altogether, these observations may indicate that Hrs depletion impairs the myogenic transcriptional program as a consequence of altered EGFR degradation causing an upregulation of the MEK/ERK pathway, although one cannot exclude the involvement of others RTK or membrane receptors from different classes.

Indeed, Hrs was previously found to regulate the trafficking and/or degradation of different classes of membrane receptors in different models [41, 48, 73]. Like EGFR, the Toll-like receptor 4 (TLR4), the RTK fibroblast growth factor receptor (FGFR), or the transforming growth factor beta receptor (TGF-β R) activations have all been described to inhibit the differentiation of myoblasts into myotubes [47, 74, 75]. Interestingly, Hrs was found to regulate the trafficking and degradation of TLR4 [76] and depletion of Hrs was responsible of the accumulation of TLR4 and its activation. FGFR downregulation was also attributed to Hrs (recently reviewed in [77]). In Hrs-depleted cells, FGFR may activate the MEK/ERK pathway as previously described [47]. Similarly, Miller et al. found that dynamic of the TGF-β signaling is dictated by receptor trafficking via the ESCRT machinery, especially via the HD-PTP factor [73]. A recent study identified a non-canonic activation of TGF-β receptor that inhibits the differentiation process via the upregulation of the MEK/ERK pathway, a phenotype that could be rescued by addition of U0126 MEK/ERK inhibitor [78]. Collectively, these data suggest that other potential membrane receptors of different classes that we did not explore could in absence of Hrs participate to the inhibitory effect of Hrs depletion on muscle differentiation.

We also observed that Akt2 expression and activity were decreased in Hrs-KD cells. Interestingly, Akt2 signaling is upregulated during myogenic differentiation and promotes differentiation via increasing myogenin transcription [2, 79]. Akt2 deficiency induced a downregulation of MEF2A expression in cardiomyocytes and skeletal muscles [80, 81], thus correlating well with our data and indicating that alteration of Akt2 expression and activity upon Hrs depletion very likely participates to the differentiation defect phenotype by altering the transcription of mef2. In addition, FOXO1 needs to be inhibited by phosphorylation in the early steps of differentiation to allow an efficient myogenesis (reviewed in [54]). FOXO1 phosphorylation by Akt2 was shown to prevent its nuclear entry [82]. Consistently with Akt2 downregulation upon Hrs depletion, we found increased FOXO1 nuclear localization. Collectively, FOXO1 activation is well correlated with Irs1 trafficking defect and the global decrease of Akt2 and highlights Irs1/Akt2/FOXO1 as an additional altered signaling pathway that participates to the early differentiation defect observed in Hrs-KD cells.

Akt2 activation during myoblasts differentiation is mediated by IGFR and Irs1 and is regulated by the small GTPase Rab5a that coordinates the recruitment of Irs1 and IGFR on early endosomes [10, 53]. Our observation of a lack of Irs1 enrichment in enlarged early endosomes in absence of Hrs, could provide a mechanism by which Hrs depletion could impair Akt2 signaling although the mechanisms by which Hrs depletion could negatively alter the recruitment of Irs1 to early endosomes remains to be elucidated.

McMillan and Quadrilatero (2014) demonstrated that efficient autophagy is necessary for the efficient myogenesis [14]. Hrs was shown to impair the autophagic flux and the degradation of ubiquitinated proteins [28–30, 38]. Similar results were observed for Tsg101 [29]. In the present study, we found that both Hrs and Tsg101 depletion impairs the autophagic flux in C2C12. Interestingly, Tsg101 depletion impaired the autophagic flux with similar efficiency if not higher compared to Hrs thus suggesting that part of the differentiation defect observed in Hrs and Tsg101 could be due to a common alteration of the autophagy process.

During the course of this study, the impacts of Hrs and Tsg101 depletions on the process of differentiation were compared. While Hrs depletion has a strong effect on the differentiation, absence of Tsg101 has a much milder effect. Here we found that Tsg101 depletion does not recapitulate the defects observed upon Hrs depletion excepted in the modulation of the autophagic flux. Indeed, absence of Tsg101 did not affect EGFR trafficking/degradation and consequently did not activate the MEK/ERK pathway. Moreover, Tsg101 depletion did not affect Akt2 signaling nor upregulated FOXO1. These data suggest that despite Tsg101 and Hrs belong to the same machinery, their role during the myogenesis differ. Absence of impact on this signaling in Tsg101-KD cells is contrasting to what was previously observed in the cancer HeLa cellular model [23, 24]. Conversely, Zhang et al. observed that ERK1/2 phosphorylation was decreased upon Tsg101 depletion in MCF7 cells [83]. These apparent contrasting data could eventually be explained, in part, by the different cellular models used in these studies. However, it should be emphasized that C2C12 differentiation is a highly complex regulated process mediated through the paracrine/autocrine release of positive and negative myogenic factors as well as controlled at different levels by several myogenic transcription factors that act as terminal effectors of different interconnected signaling cascades. This high level of complexity might explain the differences observed between the HeLa cellular model and the C2C12 myoblasts/myotubes system.

As stated before, we found that Tsg101 depletion mildly impairs the differentiation process as compared to Hrs. Many hypotheses can explain this observation. The first one could suggest that silencing of Tsg101 could be less efficient compared to that observed for Hrs. We do not think that it is the case since the impact of Tsg101 depletion on the autophagic flux was at least similar if not higher compared to Hrs depletion context. The second hypothesis is that Tsg101 depletion could be compensated, in part, by a parallel arm involving the accessory proteins Alix or Ptpn23/HD-PTP containing the Bro1 domain that can directly engage the ESCRT-III complex. As discussed above, Ptpn23/HD-PTP depletion strongly affects the process of differentiation through an Hrs-dependent mechanism. Collectively, these data highlight the key role of Hrs in the myogenesis.

The glycogen synthase kinase 3 beta (GSK3β) is a serine-threonine kinase involved downstream of multiple cellular signaling pathways. In the process of myogenesis, the active phosphorylated Y216-GSK3β acts as a negative regulator of skeletal muscle differentiation and inhibition of GSK3β by low therapeutic dose of lithium has been shown to enhance myotube formation [58]. Interestingly, Taelman et al. observed that HRS negatively controls GSK3β activity through its sequestration into the multivesicular endosomes [26]. Here, we observed increased level of active GSK3β in Hrs-depleted C2C12 cells. Furthermore, we found that GSK3β inhibition by the specific GSK3β inhibitor azakenpaullone significantly rescues the Hrs-KD differentiation defect. Interestingly, MEK1/2 was previously involved in the Y216 phosphorylation and activation of GSK3β in human skin fibroblasts [84]. This last observation could explain how Hrs depletion, through the activation of the MEK/ERK signaling pathway, could enhance GSK3β activity in the C2C12 model. Collectively, our data indicate that GSK3β is an additional signaling component dysregulated by Hrs depletion that contribute to the impaired myogenesis process phenotype.

In 1967, Meier [85] identified a spontaneous mutation named tn (for teetering) and recently identified to correspond to a single-nucleotide substitution in the Hrs encoding gene [86]. The tn mice exhibit hypokinesis, muscle weakness, reduced muscle development, significant motor deficit, functional alterations of the neuromuscular junction, and a perinatal lethality. Loss of Hrs resulted also in the accumulation of ubiquitinated proteins at the synapse. Although no investigation was specifically carried out on the role of this mutation in the muscle tissue of mutated tn mice, these studies revealed that tn mutation of Hrs results in an autosomal dominant inheritance pattern similar to that observed in human cases of amyotrophic lateral sclerosis (ALS) and Charcot-Marie-Tooth diseases. Interestingly, Oshima and colleagues also observed that forebrain-specific Hrs knockout mice display pathological phenotypes resembling those found in neurodegenerative disorders including formation of pathological p62/Ubiquitin-positive aggregates containing TDP-43, α-synuclein, or Huntingtin misfolded proteins [30]. The sporadic inclusion body myositis (sIBM) displays also the interesting feature to accumulate several pathological prion-like proteins involved in neurodegenerative disorders [87]. It would be interesting to investigate if HRS expression or trafficking could be altered in muscle tissues from sIBM-affected patients.

Interestingly, we observed a significant upregulation of Hrs protein level in tibialis anterior muscle samples of the two well characterized dystrophic mouse models Duchenne muscular dystrophy (mdx model [59];) and the muscle-specific mTOR knockout [60]. These data open new insights on the implication of Hrs in neuromuscular pathologies.

## Conclusion

Our study identifies Hrs as a master regulator of myogenesis that controls the differentiation of myoblasts into myotubes at different levels including trafficking, degradation, and activation/inhibition of signaling pathways. Hrs is required at early stages of differentiation for proper MEK/ERK and Akt2/FOXO1 signaling, in order to allow MyoD and MEF2 to induce myogenin expression and thereby myoblasts fusion into myotubes and activation of muscle late differentiation markers. We also found that GSK3β is an additional signaling component dysregulated by Hrs depletion that contribute to the impaired myogenesis.

In the future, it would be of importance to investigate whether Hrs dysfunctions might contribute to human neuromuscular disorders.

## Methods

### Antibodies

Primary antibodies were as follows: The anti-HRS/HGS antibodies (ab155539; WB 1:1,000, IF 1:100,); -MEF2A (ab32866; IF 1:500), and -GSK3β (ab32391 WB 1:1,000 and IF 1:100) were from Abcam. The anti-TSG101 (sc-7964; WB 1:500) were from Santa Cruz Biotechnology. The anti-EEA1 (CST3288; IF 1:100); -phospho-ERK1/2 (Thr202, Tyr204) (CST3179; WB 1:2,000); -total-ERK1/2 rabbit (CST9102; WB 1:1,000); -LC3 (CST2775; WB 1:1,000 and IF 1:100), -GAPDH (CST2118; WB 1:10,000), -total-AKT2 (CST2964; WB1:1,000), -phospho-AKT2-Ser474 (CST85995; WB 1:1,000); -FOXO1 (CST2880; WB 1:1,000 and IF 1:100); -Ubiquitin (CST14049; WB 1:1,000); -IRS1 (CST2382; IF 1:100); Histone H3 (CST4499S; WB 1:5,000); Rab7 (CST95746; IF 1:100), Rab5 (CST46449; IF 1:100), and phosphor-EGFR-Tyr1068 (D7A5, CST3777S; WB 1:000; IF1:100) were from Cell Signaling Technology. The anti-SQSTM1/p62 (GP62-C; WB 1:4,000 and IF 1:200) was from Progen and the anti-myogenin (F5D; WB 1:200, IF1:100) and the anti-myosin heavy chain (MHC) antibody (MF20 hybridoma cell culture supernatant) were from Developmental Studies Hybridoma Bank (DSHB). The anti-clathrin heavy chain (610499; IF 1:100) and the anti-pY216GSK3β (612312; WB 1:1000) were from BD Biosciences. The anti-KDEL (NBP1-97469; IF 1:100) were from Novus Biologicals. The anti-MyoD (MA1-41017; WB 1:1000 and IF 1:100), -EGFR (PA1-1110; WB 1:1,000), -PTPN23 (PA5-76478; WB 1:1000) were from Thermo Fisher Scientific. The anti-GM130/GOLGA2 (NBP2-53420; IF 1:200) was from Bio-techne.

Secondary antibodies are as follows: For Western blotting, the anti-mouse (NA931V) and anti-rabbit (NA934V) labeled with peroxidase (GE Healthcare UK) were used at 1:10,000.

For immunofluorescence, the goat anti-mouse dylight-488 (115-486-072), goat anti-mouse dylight-549 (115-506-072), the donkey anti-mouse Alexa-Fluor 647 (715-606-151), and the donkey anti-rabbit rhodamine (TRITC) (711-025-152) were purchased from Jackson Lab. The donkey anti-rabbit Alexa-Fluor 488 (A21206) was purchased from Molecular probe. All these secondary antibodies were used at 1:1000. Antibodies for flow cytometry are as follows: CD34-FITC (#11-0341-82); Ly-6A/E (Sca-1)-PE (#12-5981-82); CD45-PE (#12-0451-82); and CD31 (PECAM-1)-PE (#12-0311-82) were purchased from Thermo Fisher Scientific and were used as previously described by the manufacturer.

### Drugs and compounds

The inhibitor of MEK1/2-ERK1/2 U0126 (U120) and the GSK3β inhibitor 1-azakenpaullone (A3734) were used at 3 μM in DMSO and were purchased from Sigma-Aldrich. The cycloheximide (ref 239763-1GM) from Millipore was used at 10 μg/mL in DMSO.

### Constructs

The psPAX2 plasmid is a 2nd generation lentiviral packaging construct encoding the HIV-1 Gag, GagPol, Tat, and Rev accessory proteins, and the pMDG2 plasmid encoding the vesicular stomatitis envelope glycoprotein (VSVg) was kindly provided by Didier Trono (EPFL). The pLKO1 constructs encoding small hairpin RNAs (shRNA) directed against murine Hrs (Hrs#1 TRCN0000313945;  Hrs#2  TRCN0000314016  and  Hrs#3TRCN0000088688),  Tsg101  (Tsg101#1 TRCN0000054603;  Tsg101#2TRCN0000054604,  Tsg101#3 TRCN0000054607) were from Sigma-Aldrich (Mission shRNA plasmid DNA).

The HIV-psi-LVRU6GP encoding the shCT (CSHCTR001-1) or shPtpn23/HD-PTP#1 et #2 (MSH025913-1 and MSH025913-2 respectively) were kindly provided by Giampetro Schiavo and were previously described in [88].

The negative pLKO1 shRNA control (shCT) as well as the  human  HRS-shRNAs  constructs (huHRS#1TRCN0000037894; huHRS#2 TRCN0000037895 and huHRS#3 TRCN0000037896) were kindly provided by Clotilde Thery and were previously described in [89].

The HIV-1 pLv-hTERT-CDK4 for human primary myoblast immortalization bearing the expression cassettes encoding the HTert and CDK4 proteins and the puromycin resistance was from CloneSpace LLC (SKU: CS1031).

The pCDNA3.1-Myc-Empty and pCDNA3.1-Myc-murine-Hrs were kindly provided by Mark von Zastrow [19]. The pEGFPC1 encoding the GFP-human-HRS construct was kindly provided by Dr daSilva and was previously described in [90].

### Cell lines and primary muscle cells

The C2C12 skeletal myoblast cell line (ATCC CRL-1772) was kindly provided by S.Rome (IGFL, ENS-Lyon, France) and was previously described in [91]. C2C12 were grown in the growth medium (GM) Dulbecco’s modified Eagle’s medium containing glutamax/pyruvate/glucose (DMEM Gibco) supplemented with 10% (v/v) of fetal bovine serum (FBS, Gibco) and 1% penicillin streptomycin (P/S) and maintained at 37 °C under 5% CO_2_. Myoblast to myotube cell differentiation was induced by shifting the cells into the differentiation medium (Diff; DMEM supplemented with 2% of horse serum HS, Gibco and 1% P/S).

Primary muscle cells were isolated as previously described [92]. Briefly, mice were sacrificed and hind limb muscles were recovered and enzymatically dissociated with collagenase B (11 088 831 001, Roche) and Dispase (04 942 078 001, Roche) for 30 min at 37 °C. Digestion was stopped by the addition of FBS and the mixture was filtered through a 70-μm cell strainer (15-1070 BioLogix) and mildly centrifuged at 350×*g* (5804 r, Eppendorf) for 7 min at 4 °C. The cell pellet was resuspended in phosphate buffer (1× PBS) containing 2% FBS and incubated with appropriate antibodies (α7 integrin-647, CD34-FITC, CD45-PE, CD31-PE, Sca1-PE) at 4 °C during 45 min protected from the light. Cells were centrifuged at 350×*g* for 5 min at 4 °C and resuspended in phosphate buffer containing 2% FBS and passed through 30 μm CellTrics strainer. Cells were FACS sorted with FACSAria (ANIRA, Gerland, Lyon, France). The primary muscle cells are the CD31/CD45/Sca1^−^ CD34/α7integrin^+^ population. Primary muscle cells were grown into DMEM F/12 supplemented with 20% of HS, 1% PS, and 2.5 μg/mL of FGF and dishes coated with Matrigel (#354234, Corning). Differentiation of primary muscle cells was induced by shifting the growth medium to DMEM F/12 with 2% of horse serum (HS) and 1% P/S. The human embryonic kidney cell line (HEK293T) was obtained from Généthon (Evry, France) and were cultured in DMEM-Glutamax supplemented with 10% (v/v) FBS and P/S.

The human Y711i immortalized myoblast cell line was obtained as follows. Quadriceps muscle biopsy from a 64-year-old male was obtained from the CBC (Centre de Biotechnologie Cellulaire, Groupement Hospitalier Est, Bron France). Muscle stem cells were extracted, sorted, and tested for their myogenicity as previously described [93]. Cells were then expended in proliferating KMEM medium (1 volume of M199, 4 volumes of Dulbecco’s modified Eagle’s medium (DMEM), 20% fetal bovine serum (v/v), 25 μg/mL Fetuin, 0.5 ng/mL β-FGF, 5 ng/mL EGF, 5 μg/mL Insulin from Gibco BRL). The Y711 primary cell immortalization was carried out by transducing cells with the HIV-1 lentivector encoding the HTert and CDK4 overnight in KMEM culture medium. One day after transduction, cells were passaged and cultured in presence of puromycin (1 μg/mL) for selection for 3–4 days until the death of non-transduced primary myoblast control cells was completed. Once puromycin was selected, transduced myoblasts were cultured in KMEM-D medium (KMEM medium with dexamethasone 0.2 μg/mL) in absence of puromycin. After 1 week of culture in KMEM-D medium, immortalized myoblasts can be cultured for a long term and were named Y711i (i for immortalized) myoblast. Differentiation of Y711i myoblasts into Y711i myotubes was carried out in free DMEM or free DMEM+insulin (10 μg/mL) for 4–5 days.

### RNA interference and lentivector vector production

The lentiviral vector particles were produced by transient transfection of the packaging construction plasmid HIV-1 psPAX2, a minimal genome (HIV-1 pLKO1) bearing the expression cassette encoding the shCT control (shCT) or against HRS/Hrs and Tsg101 components or the HIV-psi-LVRU6GP encoding the shCT and sh-Ptpn23/HD-PTP (#1&#2) and the plasmid encoding the VSV-G-envelope expressing construct pMDG2 (DNA ratio 8:8:4 μg of the respective plasmids) into HEK293T cells (4 × 10^6^ cells plated 1 day before transfection) by the calcium phosphate method [94]. At 48 h post-transfection, virus-containing media was collected, centrifugated at 3000×*g* for 5 min (Multifuge X1; Thermo Fisher Scientific) and filtrated (0.45 μm filter, Millipore). Viral particles were normalized using the qPCR Lentivirus Titration Kit from abm (LV900) or by radioactive Reverse Transcriptase RT test and titrated on C2C12, primary muscle cell, or human primary Y711 cells for the lentivector encoding the hTERT-CDK4 cassette. Transduction of C2C12, primary muscle cells, or Y711 human myoblasts were carried with different MOI in 6-well plates for C2C12 (80,000 cells/well) or 100-mm dishes for primary muscle cells (200,000 cells/dish) or in 12-well plates (80,000 cells plated 1 day before) in presence of 6 μg/mL of polybrene overnight.

One day after transduction, cells were passaged and cultured in presence of 1 to 4 μg/mL puromycin (P8833, Sigma-Aldrich) for 2 to 3 days. Efficiency of the knockdown was monitored by Western blotting using the anti-HRS and anti-TSG101.

Alternatively, to control Irs1 antibody specificity, Irs1 was downregulated using small RNA siIrs1 ON-TARGET plus Mouse Irs1(L-040503-02-0005) or ON-TARGET plus non-targeting pool (D-001810-10-05) as negative control from Dharmacon (Horizon). siRNAs were transfected using the Lipofectamine RNAi max reagent (Thermo Fisher Scientific) according to the procedure depicted by the manufacturer.

### Transfection of C2C12 cells

C2C12 myoblasts were transfected with 2–4 μg of DNA and with 10 μL of Lipofectamine 2000 reagent (Invitrogen) in OptiMEM according to the manufacturer protocol and differentiated 7 h or 1 day later. Cells were recovered 4–6 days after transfection for further analysis.

### Rescue experiment

Rescue experiments were carried out using the Hrs#3TRCN0000088688 shRNA that targets the 3′ untranslated region of the murine Hgs mRNA. For this purpose, C2C12 shHrs#3 selected cells were transfected using the non-targeted shHrs pcDNA3.1-Myc-murine-Hrs encoding construct or the pcDNA3.1-Myc-Empty as negative control. Transfected cells were then placed in Diff for 96 h and differentiation of myoblasts into myotubes was assessed by confocal immunofluorescence imaging.

### Counting cells

Cells were first counted and plated. Six, 24, and 72 h after plating, cells were trypsinized and counted using a cell counting Malassez chamber.

### RNA extraction and quantitative RT-PCR

Total RNA was isolated from cultured cells grown either in 100 or in 35-mm dishes using Tri-reagent (Sigma-Aldrich) or RNeasy Mini kit (74104, Qiagen). The RNA quality was verified by agarose gel. Reverse transcriptase was performed on mRNA using 5× reaction buffer (Thermo Fisher Scientific), RevertAid H Minus Reverse Transcriptase (EP0451, Thermo Fisher Scientific), and random hexamer (Eurogentec) and Biometra thermocycler. RT-qPCR reaction using SYBR Green Mastermix (Qiagen) in the CFX-connect system (Bio-Rad) was performed on the cDNA using the oligonucleotides (sens/antisens) myogenin (CAATGCACTGGAGTTCGGTC /ACAATCTCAGTTGGGCATGG), hrs (GACAAGCTGGCACAGATACG/CTCTGCACCTCCAGGTACTC), mef2A (CAGCATTCCAGGGGAAGTAA/AATCAAAGGATAAGC), and cyclosporine B (AACTTTGCCGAAAACCACAT/GATGGCACAGGAGGAAAGAG).

The relative levels of mRNAs were calculated with the second derivative method (2^−(ΔΔCt)^ equation) and normalized to cyclosporine B expression with the Bio-Rad CFX software.

### Immunofluorescence and confocal microscopy imaging

Naïve and shCT, -Hrs, -Tsg101 C2C12, and Y711i and primary myoblasts/myotubes were grown in 6-well plates, 35-mm dishes, or on 12-mm diameter coverslips (Thermo Fisher Scientific) and fixed with 4% paraformaldehyde (PFA) in 1× PBS at room temperature for 10 min or alternatively with methanol at − 20 °C for 3 to 5 min. Cells were permeabilized with 0.2% or 0.3% Triton X-100 (Sigma-Aldrich) for 5 min at room temperature and blocked 1 h with 1× PBS containing 5% normal goat serum (NGS, Gibco). Muscle fibers were fixed with 4% PFA at room temperature for 20 min. Fibers were permeabilized with 1% Triton X-100 for 15 min and blocked for 1 h with 1× PBS containing 0.5% BSA, 2% NGS, and 0.1% Triton X-100. Cells and fibers were labeled overnight with the indicated primary antibody in 1× PBS containing 2% of BSA for cells or in blocking buffer for fibers at 4 °C. After extensive washing steps with 1× PBS 0.02% Triton X-100, cells and fibers were stained for 1 h at room temperature with secondary antibodies coupled with Alexa-Fluor 488 or 555. After washings, nuclear DNA was counterstained with DAPI (D9542; Sigma-Aldrich) for 5 min at room temperature and washed 3 times.

Images were acquired using the confocal microscope Zeiss LSM-880 (CIQLE platform from the Faculty of Medicine of Lyon, France) and analyzed using the ImageJ software (version 1.8).

### Pulse-chase experiments and colocalization analysis

ShCT, shTsg101, or shHrs cells in proliferation were preincubated with cycloheximide (10 μg/mL) for 60 min before EGF stimulation, as well as during the course of the experiment to prevent EGFR synthesis as previously described [49]. Cells were pulse-stimulated during 5 min with 50 ng/mL of EGF-488 (E13345, Thermo Fisher Scientific) and then washed with warm DMEM containing cycloheximide. After chase times (15, 30, and 45 min) were indicated, cells were fixed with 4% PFA for 10 min and immunostained as described in the “Immunofluorescence and confocal microscopy imaging” section. Colocalization between EGF-488 and EEA1 was quantified with the ImageJ software (version 1.8). To quantify colocalization, a binary mask corresponding to the overlap between the green (EGF-488) and the red (EEA1) channels was generated according to [95]. This mask corresponded to the surface of colocalization between EGF-488 and EEA1 on one confocal plane. A ratio was calculated between the surface of colocalization and the total green surface (EGF-488). Results were given as percentage of colocalization of EGF-488 with EEA1. The same procedure was used to quantify colocalization of HRS around nuclei with CHC, Rab5a, Rab7, GM130, and KDEL. In this case, only the ratio of overlapping between HRS and the target proteins was used.

### C2C12 myoblast and primary muscle cell differentiation analysis

To determine the rate of myoblast differentiation, the myogenic index was used as a morphological parameter of muscle differentiation. The number of nuclei in cells positive for the MHC staining and the total number of nuclei were counted in > 6 randomly selected fields per well. Using ImageJ software (version 1.8), the myogenic index (in %) was then calculated as: $\left(\frac{\mathrm{number}\ \mathrm{of}\ \mathrm{nuclei}\ \mathrm{in}\ \mathrm{positive}\ \mathrm{MHC}\ \mathrm{staining}}{\mathrm{total}\ \mathrm{number}\ \mathrm{of}\ \mathrm{nuclei}\ \mathrm{in}\ \mathrm{counted}\ \mathrm{fields}}\right)\times 100$.

### Immunoblotting and fractionation

Whole cell lysates were prepared using radio-immunoprecipitation assay (RIPA) lysis buffer (50 mM Tris HCl, pH 8, 150 mM NaCl, 1 mM EDTA, 1% NP-40, 0.5% Na-deoxycholate, 0.1% SDS) supplemented with protease inhibitor cocktail (Complete; Sigma-Aldrich) and phosphatase inhibitor (PhosphoStop; Roche) and centrifugated at 10,000×*g*, for 10 min at 4 °C or directly lysed in 1× sample buffer (60 mM TrisHCl buffer at pH 6.8, 2% SDS, 10% glycerol, 5% β-mercapto-ethanol, 0.025% bromophenol blue). Protein concentration was determined with the Bradford reagent (Bio-Rad Laboratories), and 5–30 μg of proteins was loaded and separated by SDS-PAGE electrophoresis gels in presence of 2,2,2-trichloroethanol (TCE, T54801, Sigma-Aldrich) for stain free gel analyzes. Gels were transferred to polyvinylidene difluoride (PVDF) membranes (Millipore, IPVH0001 0.45 μm, Millipore) by semi-dry electrotransfer apparatus (TransBlot® Turbo^TM^, Bio-Rad Laboratories) in TOWBIN buffer (25 mM Tris, 192 mM glycine, 20% methanol) or wet electrotransfer (25 mM Tris, 192 mM glycine, 20% ethanol), and rate of transfer was controlled by stain free analyzes using the ChemiDoc^TM^ Touch imaging system (Bio-Rad Laboratories) analyzer. Membranes were incubated with primary antibody in 5% bovine serum albumin (BSA) in Tris-Buffered Saline (TBS buffer; UP74004B, Interchim) overnight at 4 °C. Membranes were successively washed 3 × 5 min in TBS 0.1% Tween-20 and were next probed with horseradish peroxidase-conjugated secondary antibodies for 1 h at room temperature. After three washing steps, immunodetection was carried out using the ECL^TM^ reagent (Amersham). Western blotting revelation was carried with a ChemiDoc^TM^ Touch imaging system and quantification of immunoblots was done using the image lab software (Bio-Rad Laboratories).

For nuclear and cytosol fractionation experiments, freshly cells were recovered in Buffer A (20 mM Tris HCl, pH 8, 1 mM EDTA, 5 mM DTT, protease inhibitors, and anti-phosphatase) for 15 min at 4 °C and then lyzed using a dounce tissue grinder. Cell lysate was centrifuged at 2400×*g* for 5 min and the resulting supernatant was recovered as the cytosolic fraction, whereas pellet corresponding to nuclei fraction was lyzed in Buffer B (20 mM Tris HCl, pH 8, 20% glycerol, 420 mM NaCl, 1.5 mM MgCl_2_, 0.2 mM EDTA, 0.5 mM DTT, protease inhibitors, and anti-phosphatase) for 20 min and sonicated. The lysate was centrifuged at 9600×*g* for 15 min, and the resulting supernatant was recovered as the nuclei fraction. Cytosolic and nuclear fractions were analyzed by Western blotting as described above.

### EGFR degradation

shCT, shHrs, and shTsg101 cells were preincubated for 1 h in growth medium in presence of 10 μg/mL of cycloheximide. Cells were then cultured in growth medium in presence of cycloheximide (10 μg/mL) and with EGF Recombinant Human protein (50 ng/mL; Gibco) for 15, 60, and 120 min. After these time points, cells were recovered and analyzed by Western blotting.

### Quantification and statistical analysis

All experiments were performed at least three independent times. All images analysis has been quantified with the ImageJ software (version 1.8). The number of individual experiments and the number of cells or different organelles analyzed are indicated in the figure legends. We tested our datasets for normal distribution and chose an appropriate test accordingly using Prism 6.0 (GraphPad Software). Data are given as mean ± SEM. The nonparametric two-sided *U* test (Mann–Whitney *U* test) was used to test two samples with unequal variance. For more than two samples, we used nonparametric Kruskal–Wallis test. For a multiple factorial analysis of variance, two-way ANOVA was applied. *P* values < 0.05 were considered statistically significant (shown as a single asterisk in figures, *P* values < 5%); *P* values < 0.01 were considered highly statistically significant (shown as two asterisks in figures, *P* values < 1%); *P* values < 0.001 were considered very highly statistically significant (shown as three asterisks in figures, *P* values < 0.1%) and *P* values < 0.0001 were considered extremely highly statistically significant (shown as four asterisks in figures, *P* values < 0.01%). Samples were not randomized for the experiments. No samples were excluded from the analysis.

### Mice

Control (C57BL/10) and *mdx* (C57BL/10 ScSn-Dmd*mdx*/J) mice were provided by Jackson Laboratory. The generation of control (*mTOR*^*flox/flox*^) and muscle-specific mTOR knockout (*HSA-Cre*^+^; *mTOR*^*flox/flox*^ herein called mTORmKO) mice on F6; C57BL/6 background has been previously described in [60].

Animals were provided with mouse chow and water *ad libitum* in a restricted-access, specific pathogen-free animal care facility at AniRA PBES, Lyon, France. All procedures were performed in accordance with national and European legislation on animal experimentation.

Protein extract from tibialis anterior muscle of 11-week-old mdx and 16-week-old mTORmKO male mice were prepared as described in [60].

## Supplementary Information

**Additional file 1.** **Fig. S1:** The protein level of the ESCRT-I Tsg101 increased during myoblast differentiation. **(a)** Representative Western blotting of C2C12 cell extracts collected in proliferation (Pro; lane 1) and at 7, 24, 48, 72, and 96 h of differentiation (lanes 2-6) and probed with antibodies directed against Hrs, Tsg101, myogenin antibodies and GAPDH was used as a loading control. **(b)** Quantification of the Tsg101 protein level in experiments as presented in (a). Data are presented as ratio of Tsg101/GAPDH and normalized to the starting point Pro condition. Data are presented as mean +/- SEM, n = 5 experiments. Significance was assessed using a Kruskal–Wallis test; ns not significant.

**Additional file 2.** **Fig. S2:** HRS depletion in immortalized human myoblasts negatively alters their differentiation and Hrs overexpression partially rescues differentiation. **(a)** Representative Western blotting of shCT (lane 1) and sh-huHRS#1-3 (lanes 2-4) of immortalized human myoblast (Y711i) extracts at 96 h of differentiation. Membrane was probed with antibodies directed against HRS, MHC and GAPDH was used as a loading control. **(b)** Representative immunofluorescence images of shCT and sh-huHRS#1-3 Y711i cells at 96 h of differentiation and stained with anti-MHC antibody (green) and DAPI (blue) for nuclear labeling. Scale bars, 200 μm. **(c)** Representative Western blotting of shCT and shHrs#3 C2C12 cells differentiated for 96 h and transfected one day prior differentiation with empty or Myc-Hrs encoding construct. Membrane was probed with antibodies directed against MHC, Hrs, myogenin and GAPDH was used as a loading control. **(d)** Representative immunofluorescence images of shCT and shHrs#3 C2C12 differentiated for 96 h and transfected one day prior differentiation with empty or Myc-Hrs encoding constructs. Cells were probed with anti-MHC antibody (green) and DAPI (blue) for nuclear labeling. Scale bars, 200 μm.

**Additional file 3.** **Fig. S3:** Tsg101 silencing in C2C12 delays differentiation. **(a)** Representative Western blotting of shCT (lane 1) and different shTsg101 (#1-3; lanes 2-4) C2C12 cell extracts and probed with antibodies directed against Tsg101 and GAPDH was used as a loading control. **(b)** Representative immunofluorescence images of shCT and shTsg101#1-3 C2C12 cells at 96 h of differentiation and stained with anti-MHC antibody (green) and DAPI (blue) for nuclear labeling. Scale bar, 200 μm. **(c)** Quantification of the myogenic index (differentiation index) corresponding to the number of nuclei present in MHC-positive structure as presented in (b). Data are presented as mean +/- SEM, each dot represents one image, n = 24 images from two independent experiments.

**Additional file 4.** **Fig. S4:** Hrs and Tsg101 depletion negatively alters the autophagic flux during differentiation. **(a)** Representative Western blotting of shCT and shHrs#3 C2C12 cell extracts collected in Pro (lanes 1 and 7) and at 7, 24, 48, 72 and 96 h of differentiation (lanes 2-6 and 8-12) and probed with the anti-HRS, -p62/SQSTM1, -LC3 antibodies. GAPDH was used as a loading control. **(b)** Quantification of the p62 level. Data are presented as ratio of p62/GAPDH and normalized to the starting point Pro condition as mean +/- SEM, n = 2 experiments. **(c)** Representative immunofluorescence images of shCT and shHrs#3 C2C12 probed with the anti-LC3 (green), anti-p62 (red) antibodies and DAPI (blue) for nuclear staining. White Arrows indicate accumulation of LC3 and p62 proteins in absence of Hrs. Scale bars, 20 μm. **(d)** Representative immunofluorescence images of shCT and shHrs#3 C2C12 cells at 96 h of differentiation probed with the anti-LC3 (green), anti-p62 (red) antibodies and DAPI (blue). Note the absence of myotubes and the accumulation of LC3 and p62 only in shHrs#3 cells. Scale bars, 20 μm. **(e)** Representative Western blotting of shCT and shTsg101#3 C2C12 cell extracts collected in Pro (lanes 1 and 7) and at 7, 24, 48, 72 and 96 h of differentiation (lanes 2-6 and 8-12) and probed with the antibodies directed against TSG101, MHC, p62/SQSTM1 and LC3. GAPDH was used as a loading control. **(f)** Representative immunofluorescence images of shCT and shTsg101#3 C2C12 myoblasts probed with the anti-LC3 (green), anti-p62 (red) and DAPI (blue). White Arrows indicate the strong accumulation of LC3 and p62 proteins in absence of Tsg101. Scale bar, 20 μm. **(g)** Representative immunofluorescence images of shCT and shTsg101#3 C2C12 and myotubes at 96 h of differentiation probed with the anti-LC3 (green), anti-p62 (red) and DAPI (blue). White arrows and arrowheads show strong accumulation of p62 and LC3 in undifferentiated myoblasts and myotubes respectively. Scale bar, 20 μm.

**Additional file 5.** **Fig. S5:** Comparison of Hrs and Tsg101 depletion on p62/SQSTM1 accumulation. **(a)** Representative Western blotting of cell extracts from shCT (lane 1), shTSG101 (lane 2) and shHrs (lane 3) C2C12 cells collected in proliferation and probed with the antibodies directed against Hrs, Tsg101, p62/SQSTM1. GAPDH was used as a loading control.

**Additional file 6.** **Fig. S6:** Tsg101 silencing in C2C12 doesn’t impact EGFR trafficking. **(a)** Representative Western blotting of protein extracts from shCT and shTsg101#3 C2C12 cells collected in Pro (lanes 1 and 7) and at 7, 24, 48, 72 and 96 h of differentiation (lanes 2-6 and 8-12) and probed with antibodies directed against MHC, TSG101, total-ERK1/2, p T202/Y204-ERK1/2. GAPDH was used as a loading control. **(b)** Quantification of the phosphorylated-ERK1/2 from similar experiments presented in (a). The data are presented as a ratio of pERK1/ERK1 (upper panel) and pERK2/ERK2 (lower panel) and normalized to the Pro starting point condition. Data represent mean +/- SEM, n = 2 experiments. **(c)** Left panel: pulse-chase experiments in Tsg101 depletion context: shCT and shTsg101#3 C2C12 myoblasts were stimulated for 5 min with 50 ng/mL of EGF-488 (green) and after removing unbound ligand-chased for the indicated amount of time. Cells were probed with the anti-EEA1 to visualize EE and trafficking of EGF-488/EGFR was followed through the EE pathway. Right panel: Mask of colocalization between EGF-488 and the EE marker EEA1. Scale bar, 20 μm. **(d)** Quantifications of colocalization of EGF-488 in EEA1 positive compartments: analysis shows the percentage of EGF-488 overlapping with EEA1 and establishes the endocytic trafficking of EGF ligand through the EE pathway (EEA1 compartment). Open circles correspond to shCT and black squares to shTsg101#3 conditions. The data represent mean +/- SEM. Analyses were done on 5 fields per condition, n = 3 experiments. Significance was assessed using a Mann-Whitney U test: ns not significant. **(e)** Degradation of EGFR. shCT and shTsg101#3 cells were stimulated with 50 ng/mL of EGF and with 10 μg/mL of cycloheximide to inhibit de novo synthesis of EGFR. Cells were recovered 15 (lanes 1,4), 60 (lanes 2,5) and 120 (lanes 3,6) min after stimulation and cell extracts were analyzed by Western blotting using anti-EGFR, -pY1068-EGFR, -ERK1/2, pT202/Y204ERK1/2 antibodies. GAPDH was used as a loading control. (f) Quantifications of EGFR protein level. Data represent the ratio of EGFR/GAPDH. Data are mean +/- SEM, n = 4 experiments. ns not significative.

**Additional file 7.** **Fig. S7:** Ptpn23/HD-PTP depletion reduces Hrs protein level and impairs differentiation of myoblasts into myotubes. **(a)** Western blotting of protein extracts from shCT (lane 1) and shPtpn23/HD-PTP sh#1 (lane 2) and sh#2 (lane 3) C2C12 cells collected at 96 h of differentiation and probed with antibodies directed against Hrs and Ptpn23/HD-PTP. GAPDH was used as a loading control. Note the reduction of Hrs in Ptpn23/HD-PTP-depleted cells. **(b)** Immunofluorescence images of shCT and shPtpn23/HD-PTP (sh#1&2) C2C12 cells at 96 h of differentiation and stained with anti-MHC antibody (green) and DAPI (blue) for nuclear labeling. Scale bar, 200 μm. **(c)** Quantification of the myogenic index (differentiation index) corresponding to the number of nuclei present in MHC-positive structure as presented in (b). Data are presented as mean +/- SEM, each dot represents one image, n = 16 images from two independent experiments.

**Additional file 8.** **Fig. S8:** Tsg101 silencing does not alter FOXO1 protein level. **(a)** Representative Western blotting of protein extracts from shCT and shTsg101#3 C2C12 cells collected in Pro (lanes 1 and 5) and at 24 (lanes 2,6), 48 (3,7) and 72 (lanes 4-8) h of differentiation and probed with antibodies directed against MHC, Tsg101 and FOXO1. GAPDH was used as a loading control. **(b)** Quantification of total FOXO1 protein level in experiments as presented in (a). Data are presented as ratio of FOXO1/GAPDH signal and normalized to the starting point Pro condition. Data represent mean +/- SEM, n = 3 experiments. Significance was assessed using a two-way ANOVA test; ns not significant.

**Additional file 9 Fig. S9:** Hrs protein level is upregulated in muscles of dystrophic mouse models. **(a)** Tibialis anterior muscles of Duchenne muscular dystrophy (*mdx*) mice versus control mice (CT) were isolated and protein lysates were analyzed by Western blotting and probed with anti-Hrs antibody. GAPDH was used as a loading control. **(b)** Quantification of Hrs protein level from experiments as presented in (a). Data are presented as ratio of Hrs/GAPDH and normalized to the CT. Data represent mean +/- SEM. n = 4. Significance was assessed using a Mann&Whitney test; * p < 0.05. **(c)** Tibialis anterior muscles of mTORmKO dystrophic mice versus control mice (CT; mTORflox) were isolated and protein lysates were analyzed by Western blotting and probed with anti-Hrs antibody. GAPDH was used as a loading control. **(d)** Quantification of Hrs protein level from experiments as presented in (c). Data are presented as ratio of Hrs/GAPDH and normalized to the CT. Data represent mean +/- SEM. n = 4. Significance was assessed using a Mann&Whitney test; * p < 0.05.

**Additional file 10.** Raw-data-Western blotting.

**Additional file 11.** Raw-data-Numerical.

## Data Availability

All data generated or analyzed during this study are included in this published article and its supplementary information files. Sources of data are also all included in the published article as Additional files-Raw-data-Western blotting and -Raw-data-Numerical.

## Abbreviations

ESCRT: Endosomal Sorting Complex Required for Transport
Pro: Proliferation/GrowthMedium
Diff: Differentiation/Differentiation medium
MRF: Myogenic regulatory/transcription factor
HRS/HGS-Hrs/Hgs: Hepatocyte growth factor regulated tyrosine kinase substrate
EGFR: Epidermal growth factor receptor
IGFR: Insulin growth factor receptor
RTK: Receptor tyrosine kinases
EDL: Extensor digitorum longus muscle
qRT-PCR: Quantitative reverse transcriptase polymerase chain reaction
CHC: Clathrin heavy chain
EE: Early endosome
LE: Late endosome
ER: Endoplasmic reticulum
Tsg101: Tumor susceptibility gene 101
shRNAs: Small hairpin RNAs
KD: Knockdown
MHC: Myosin heavy chain
MVB: Multivesicular body
