# Supplementary material for: The ESCRT-0 subcomplex component Hrs/Hgs is a master regulator of myogenesis via modulation of signaling and degradation pathways
Source: BMC Biol. 2021 Jul 30;19:153. doi: 10.1186/s12915-021-01091-4 (PMC8323235; doi:10.1186/s12915-021-01091-4)

Figure 1a

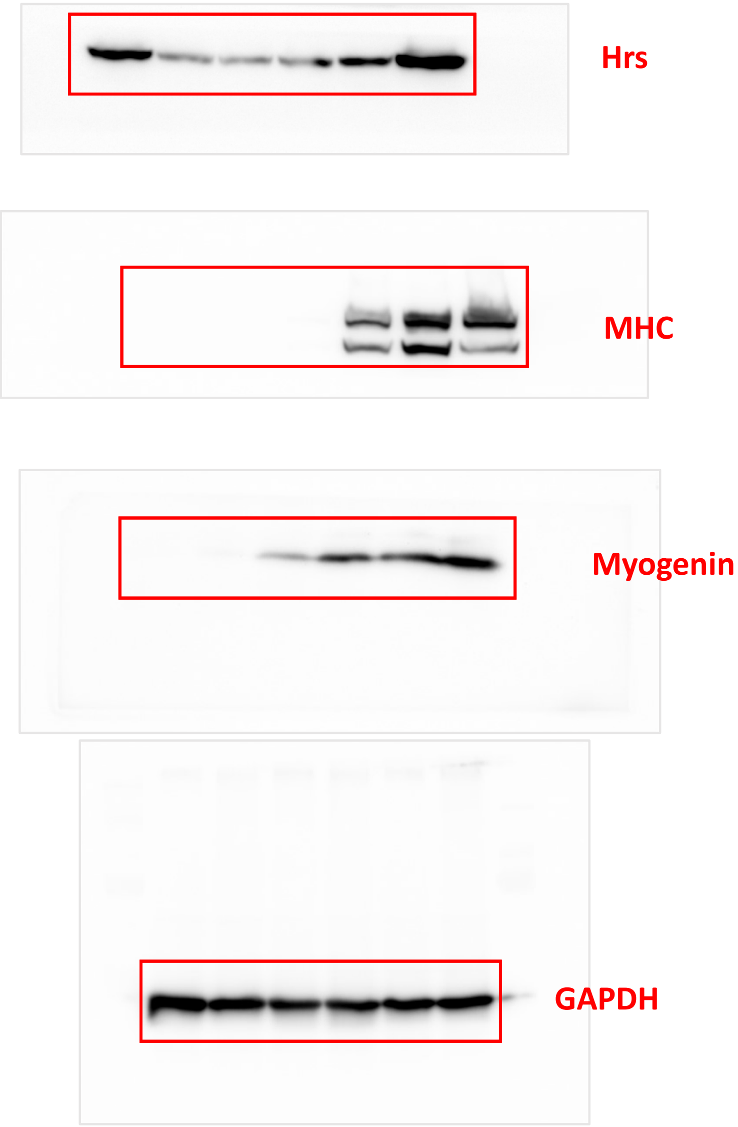

Figure 1d

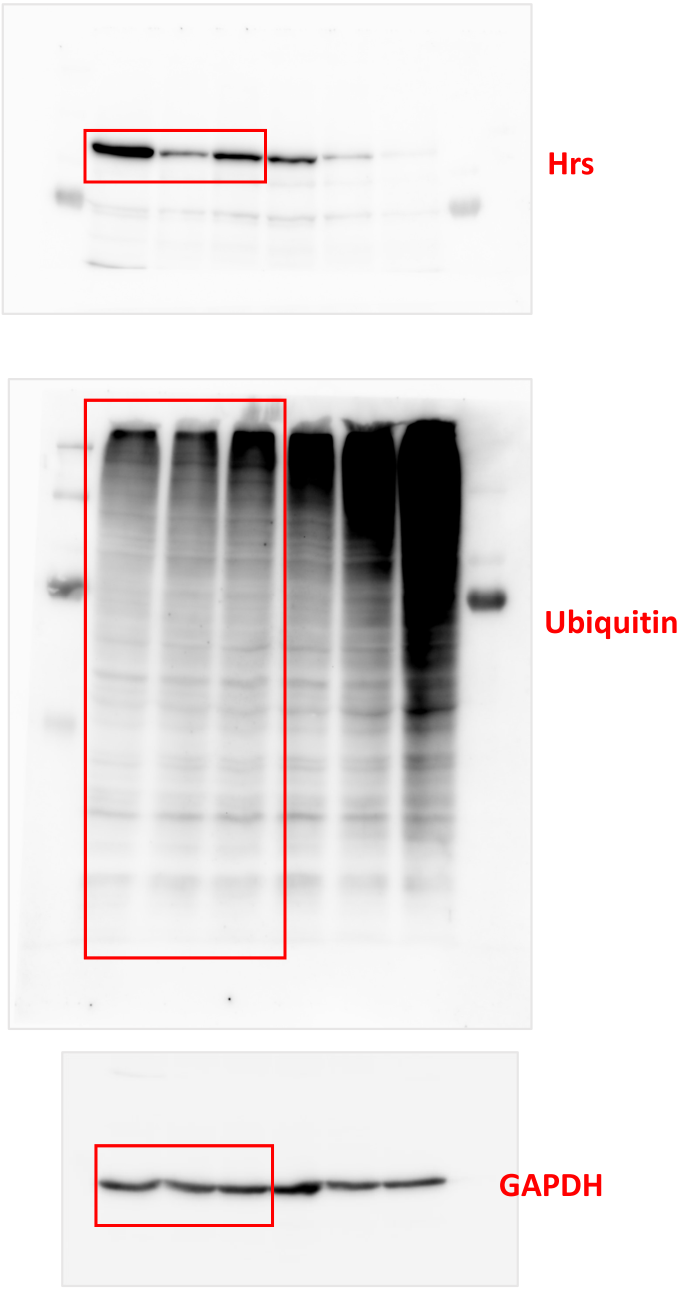

Figure 3a

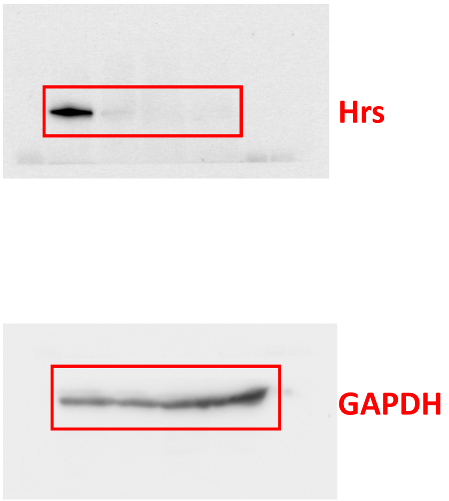

Figure 3e

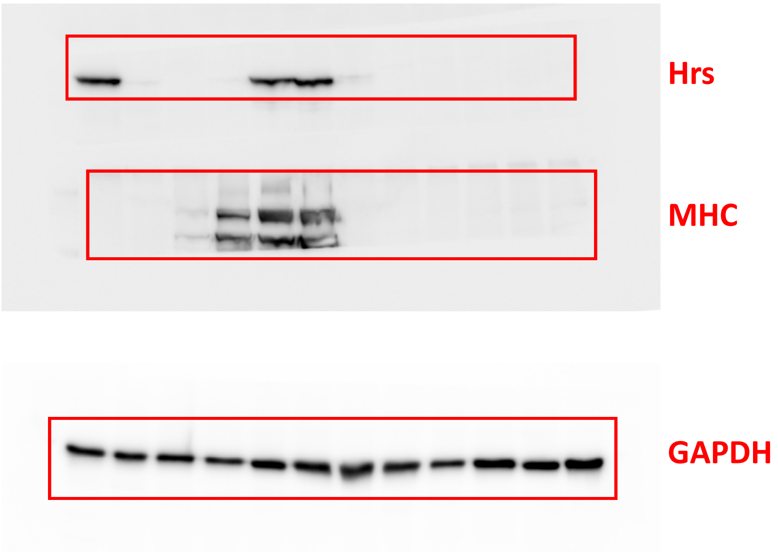

Figure 3h

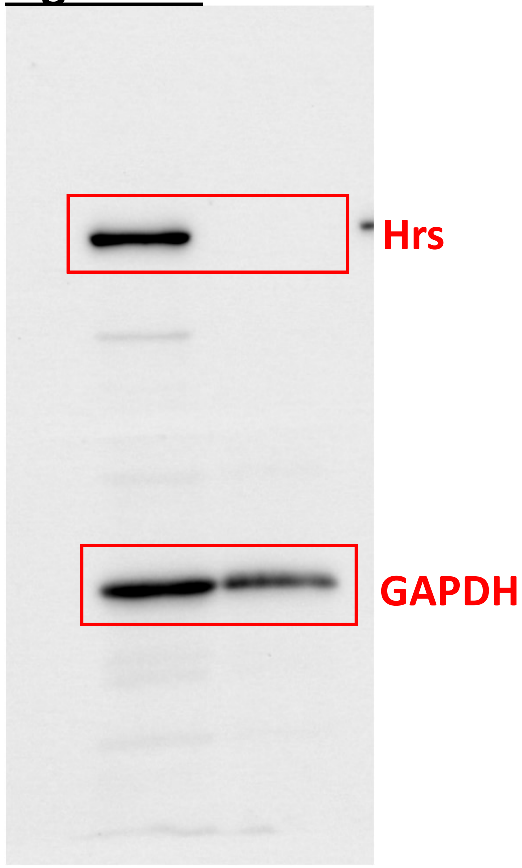

Figure 4a

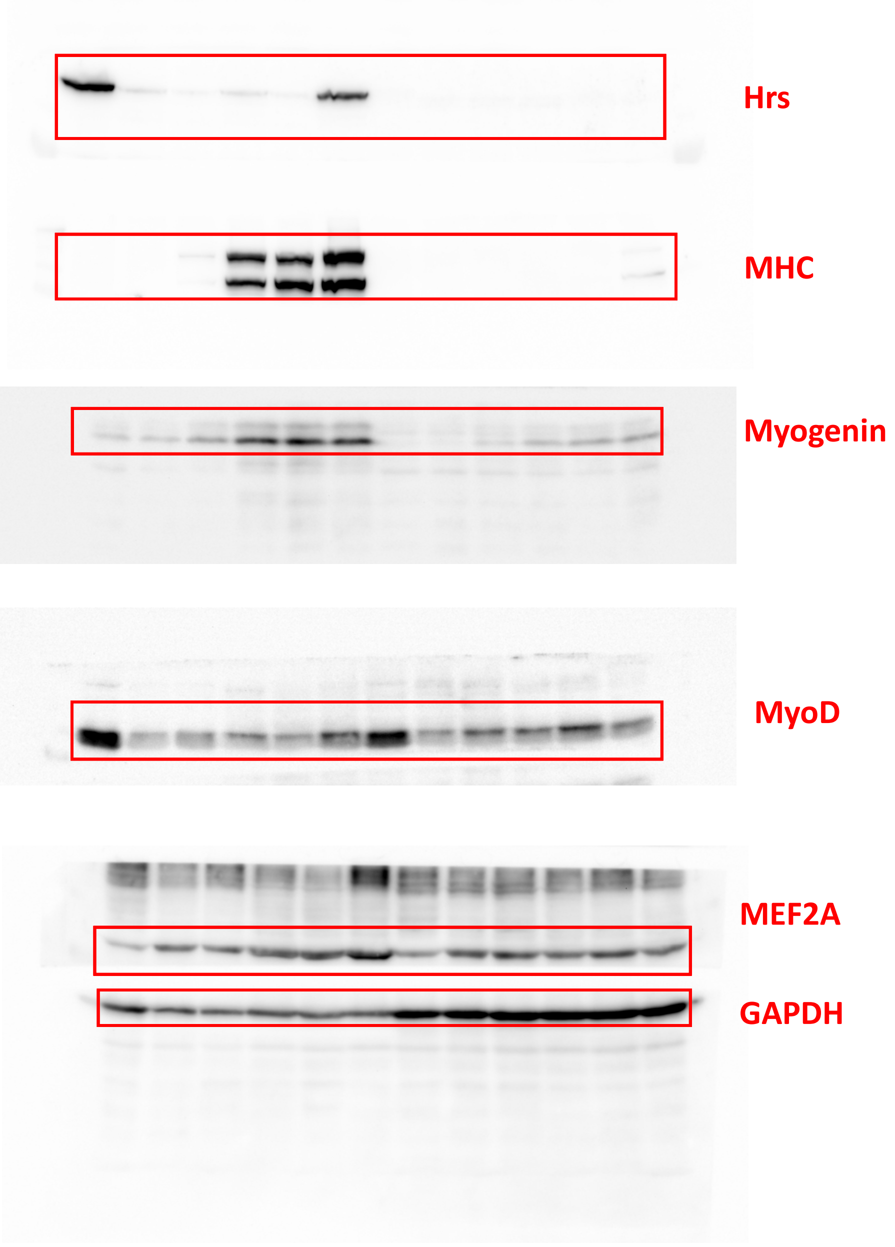

Figure 5a

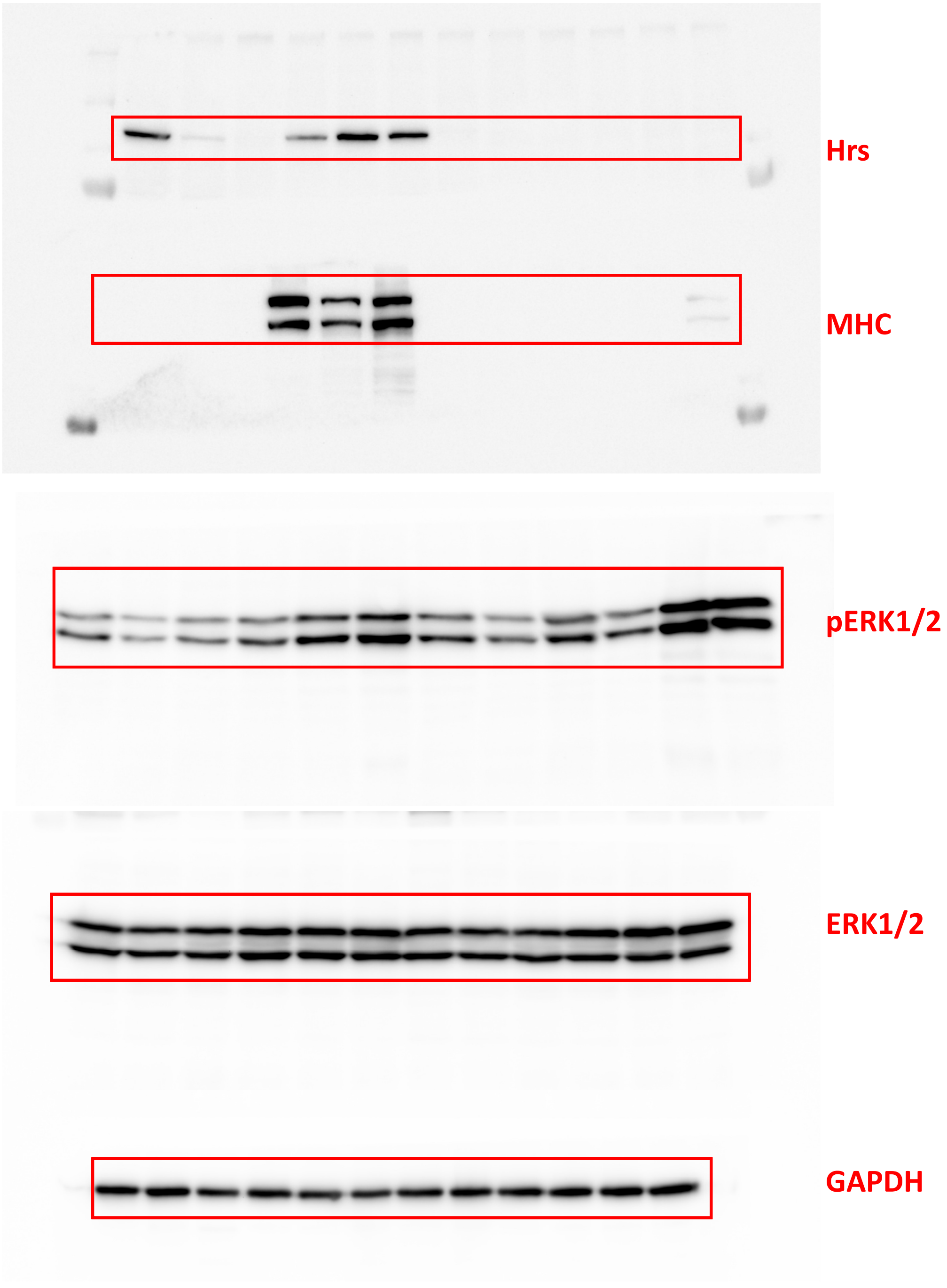

Figure 5e

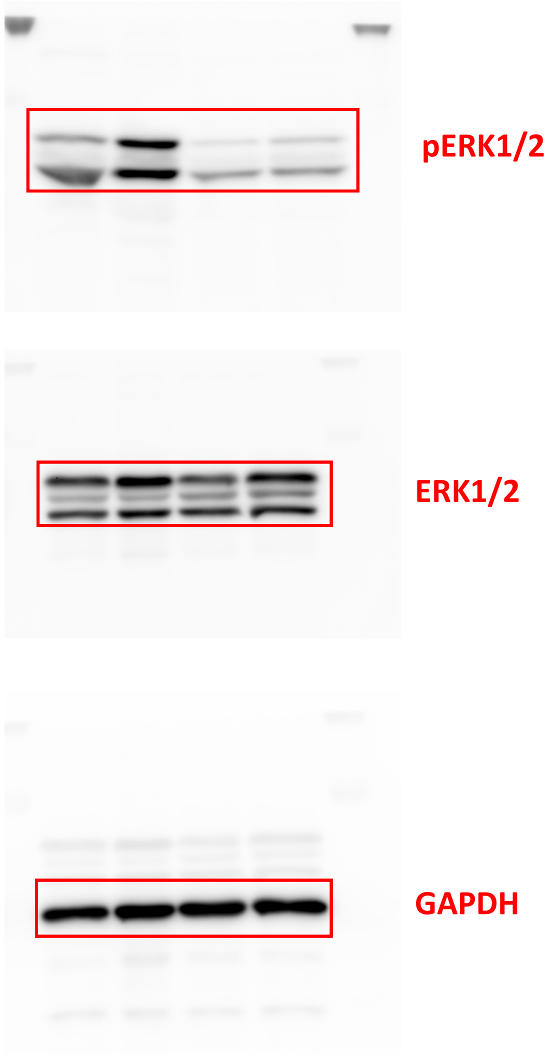

Figure 6c

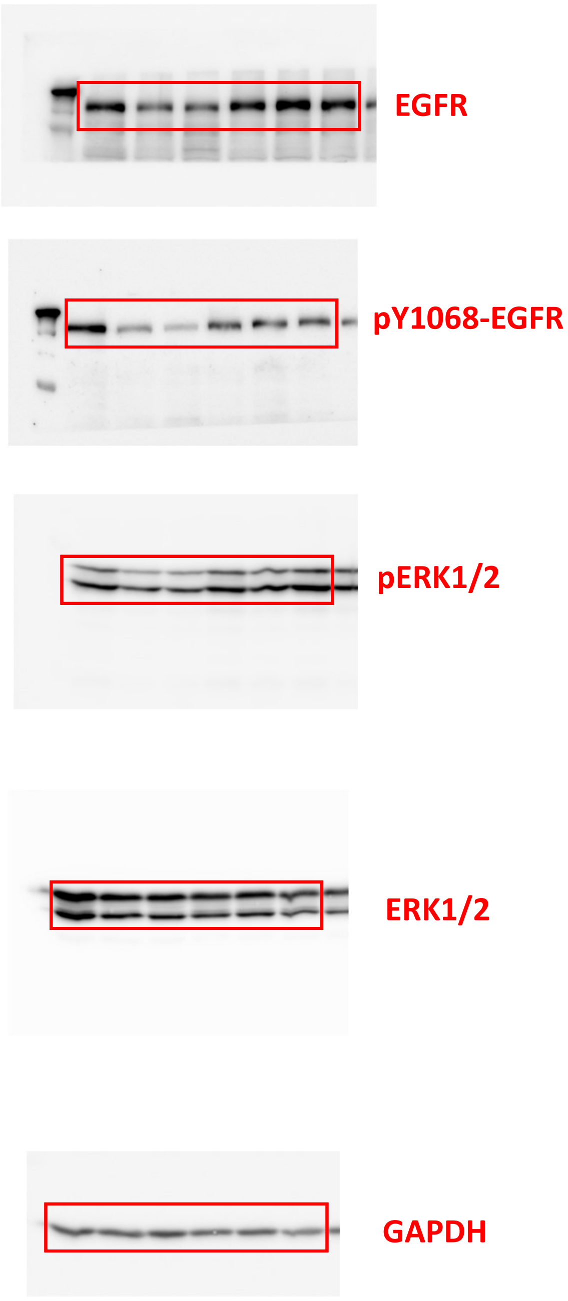

Figure 6e

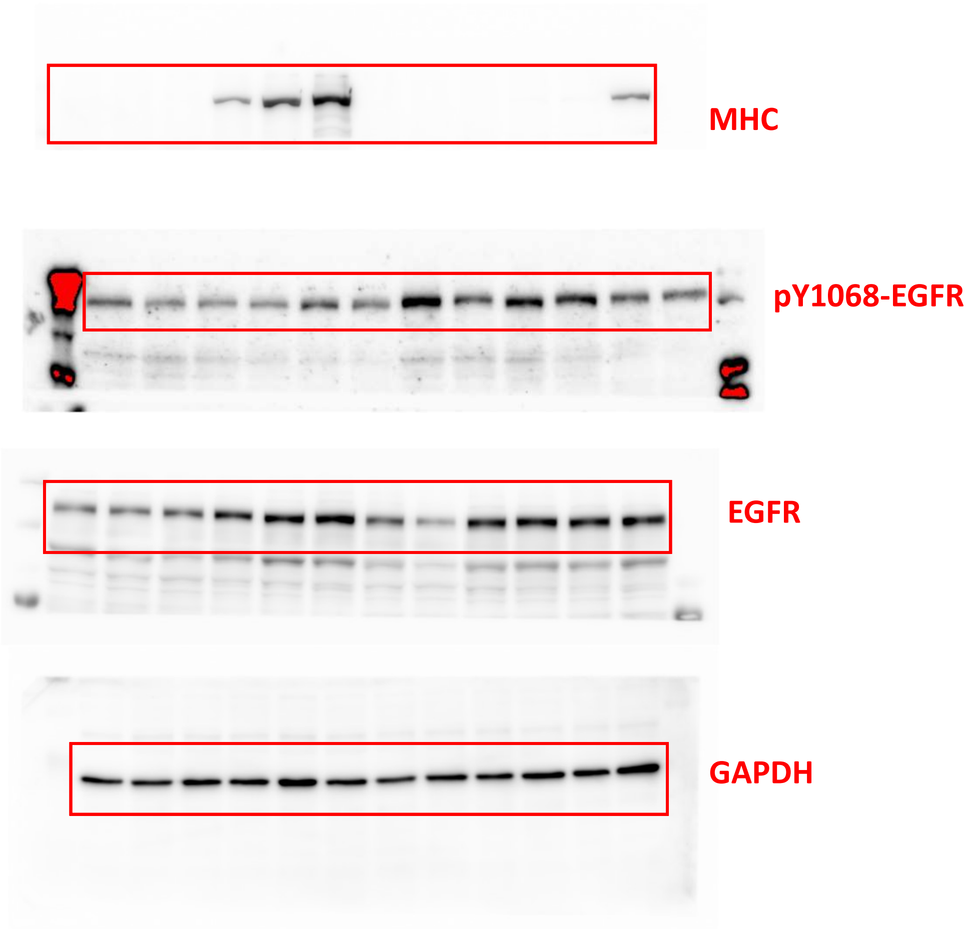

Figure 7a

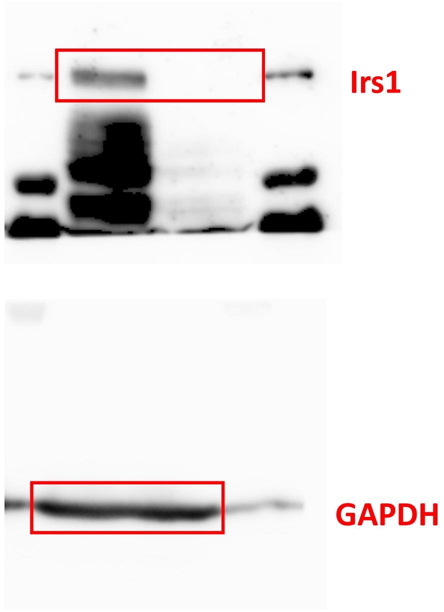

Figure 7d

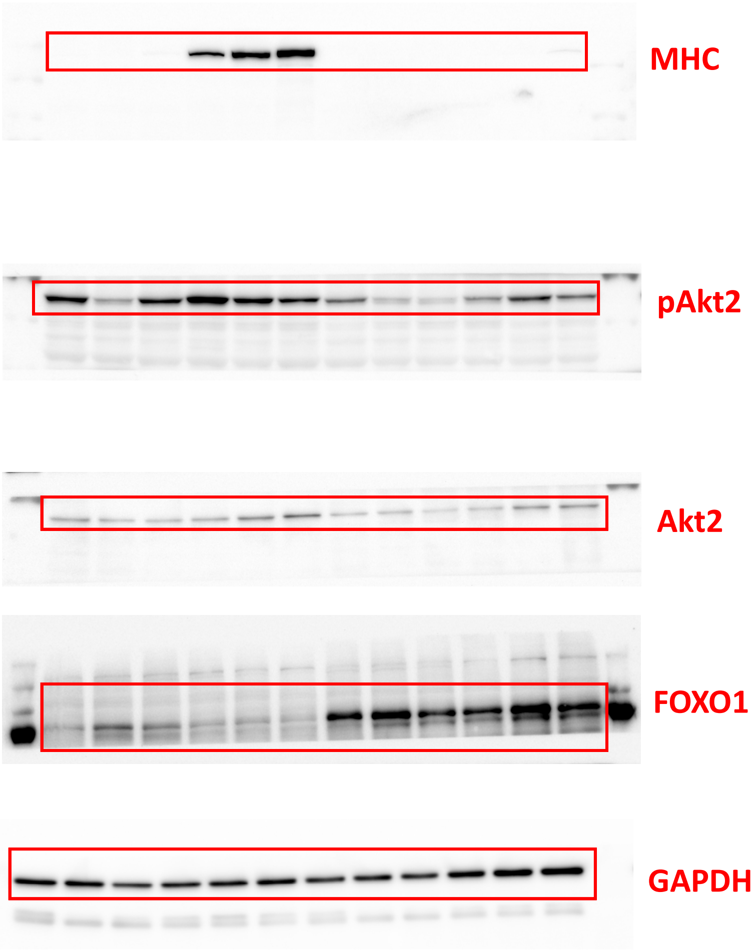

Figure 7h

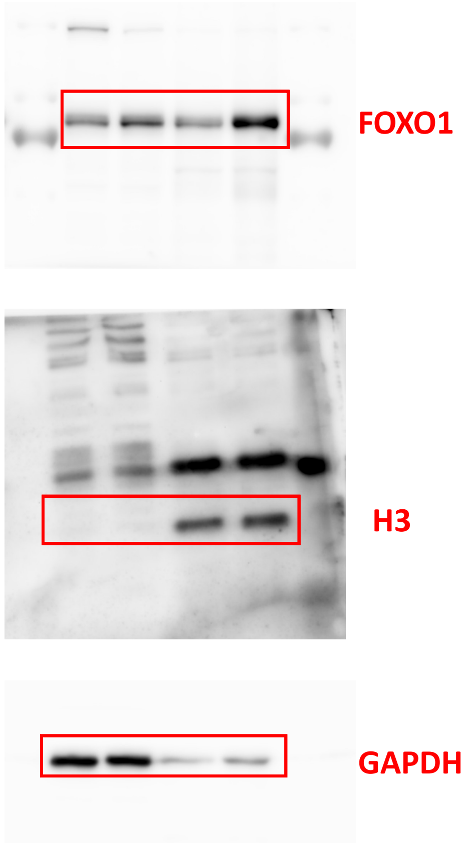

Figure 8a

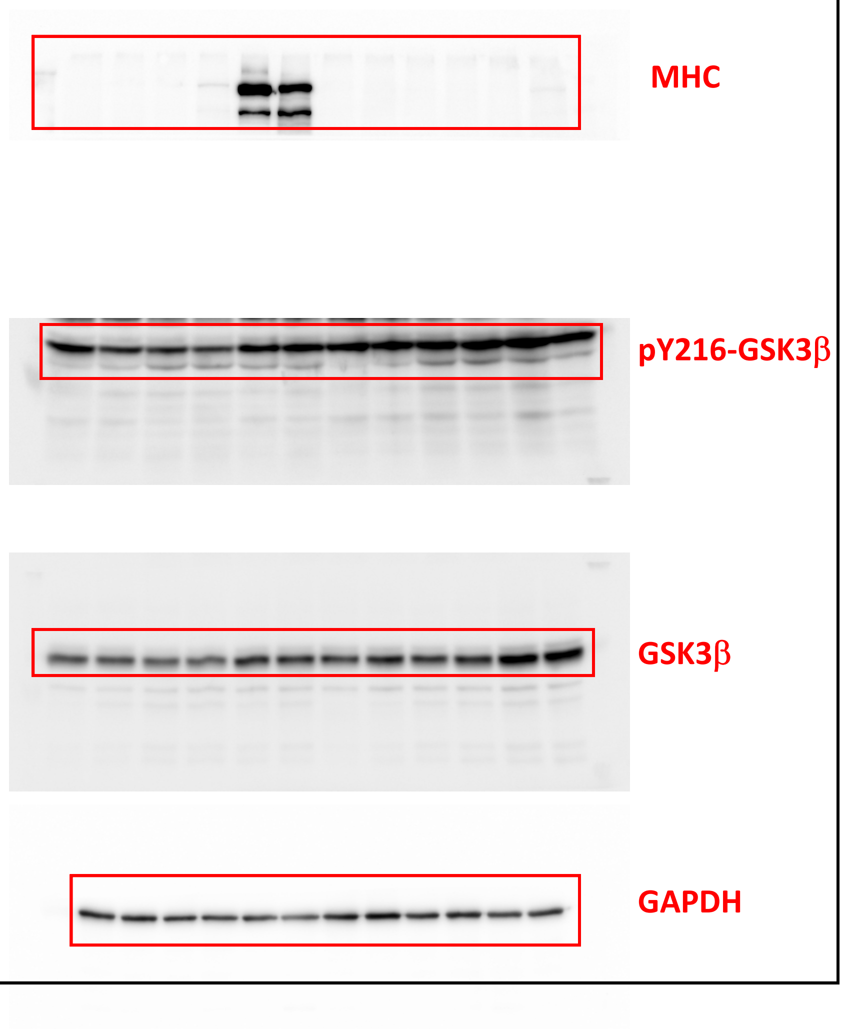

Figure S1a

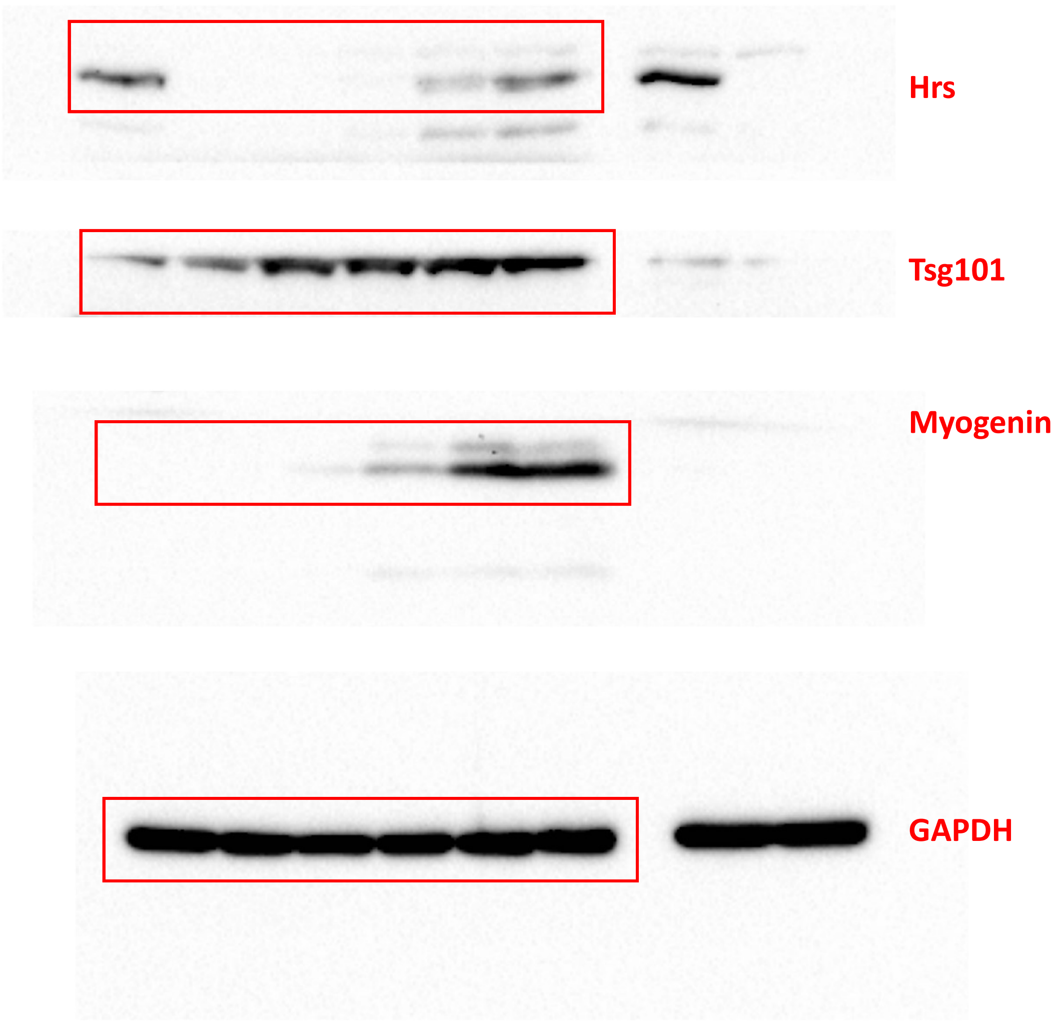

Figure S2a

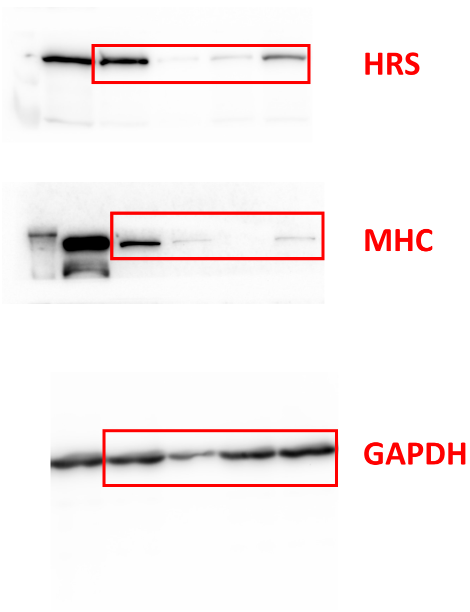

Figure S2c

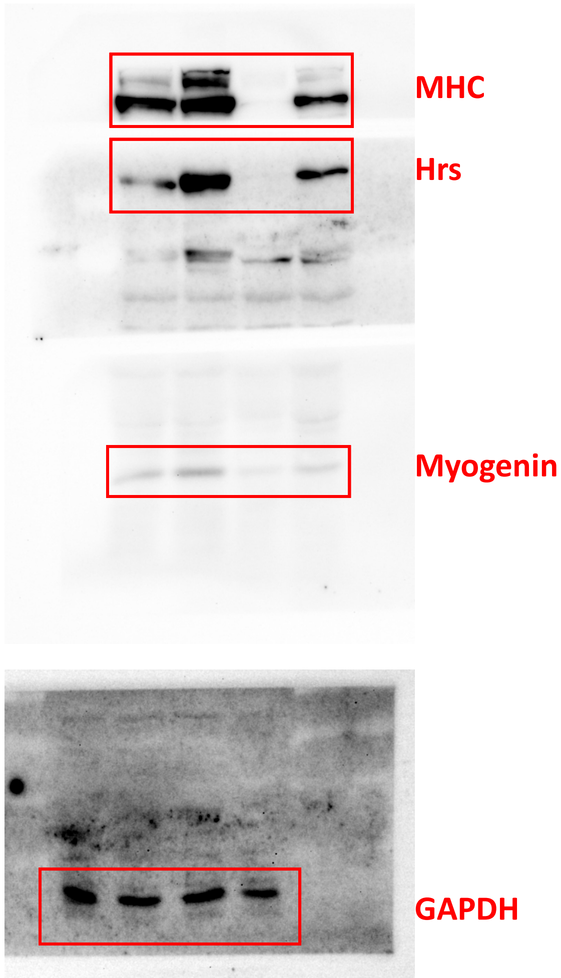

Figure S3a

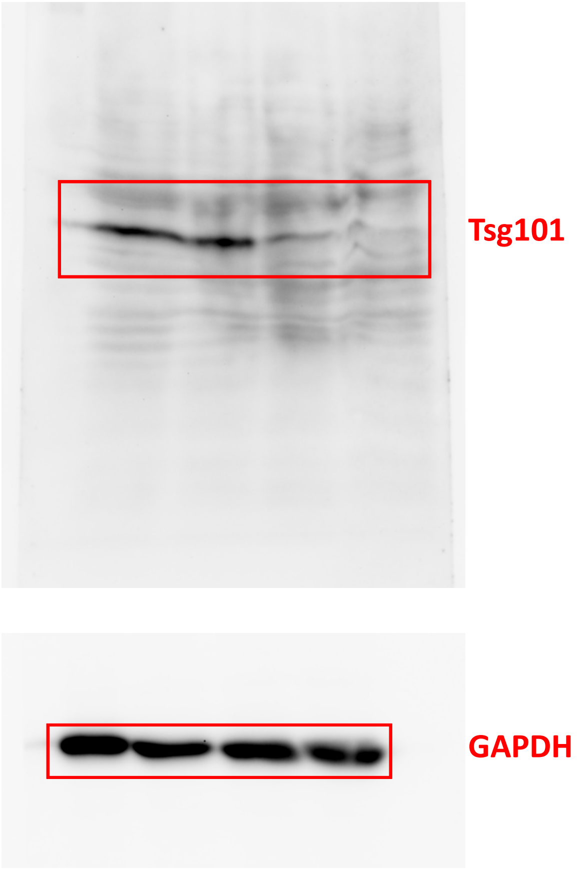

Figure S4a

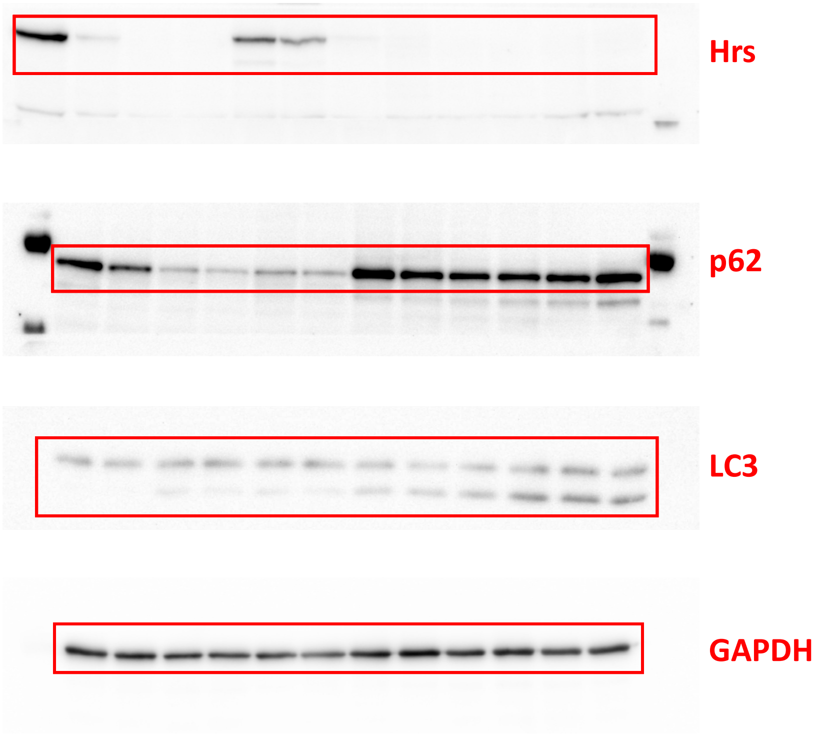

Figure S4e

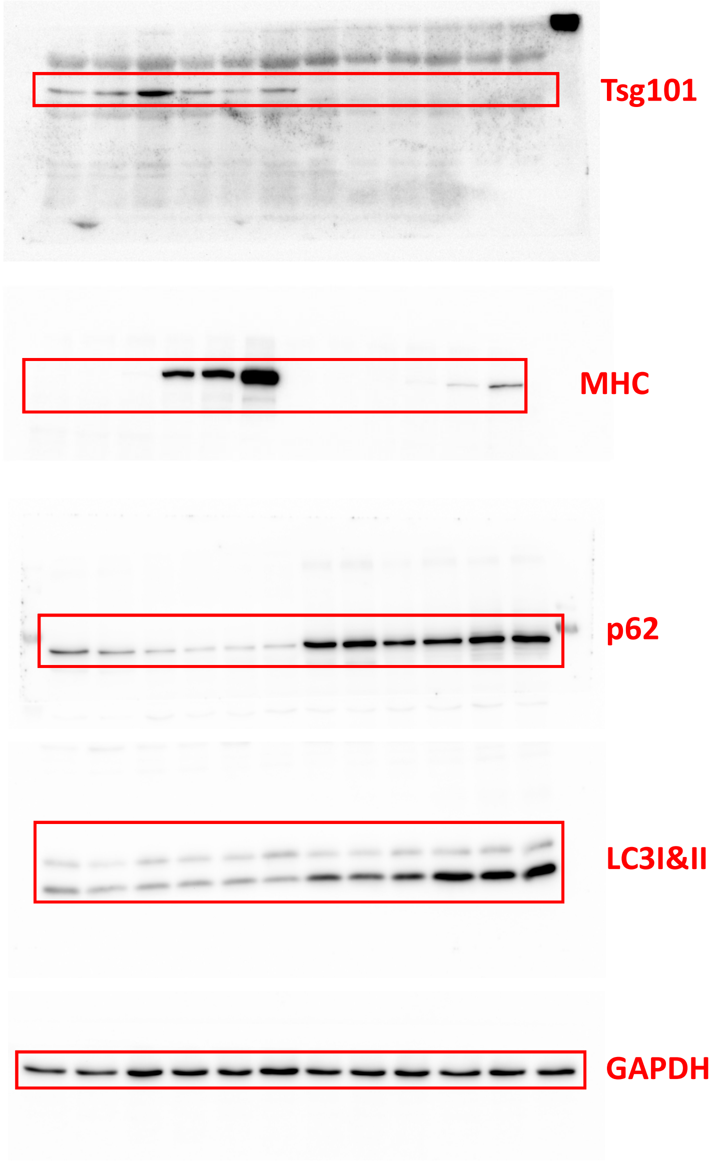

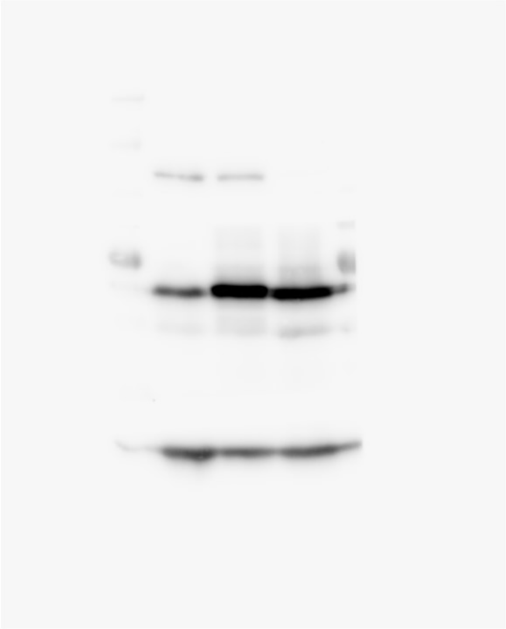

Figure S5

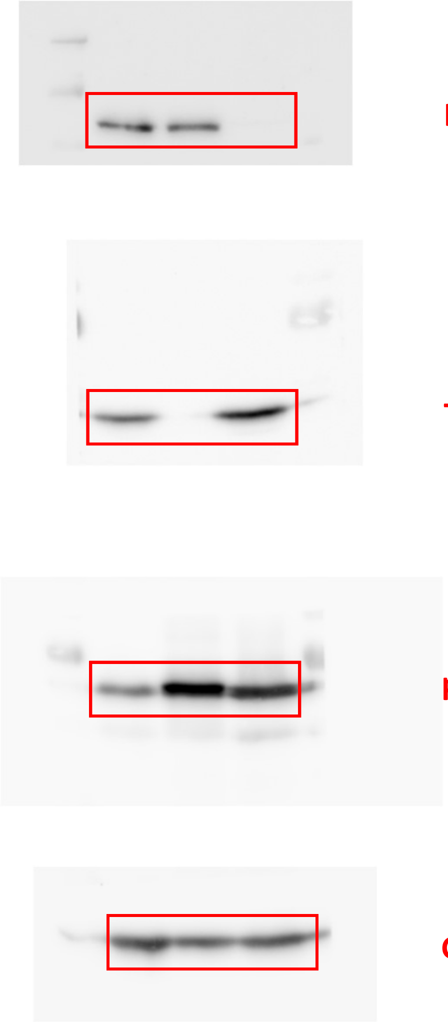

Figure S6a

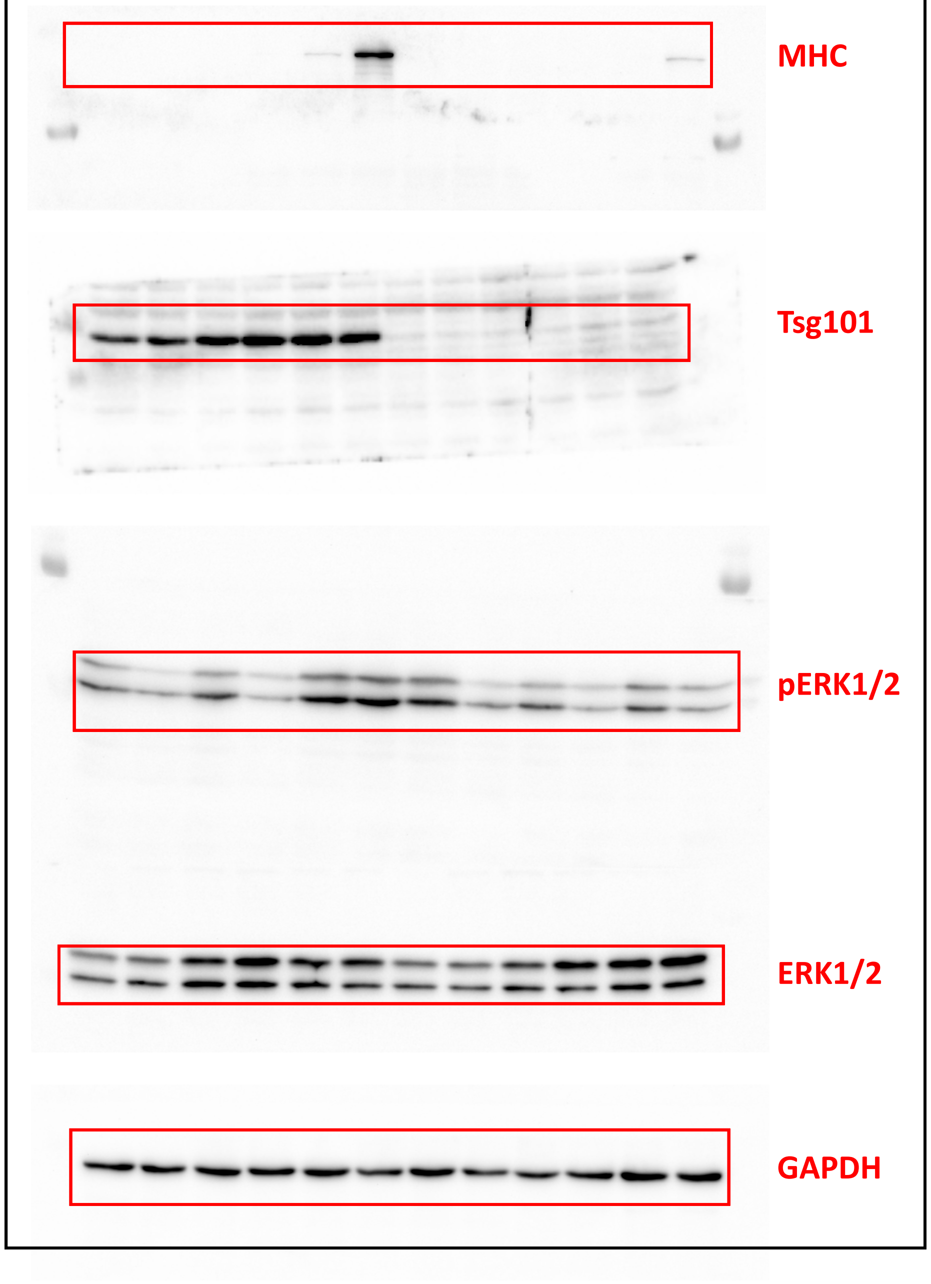

Figure S6e

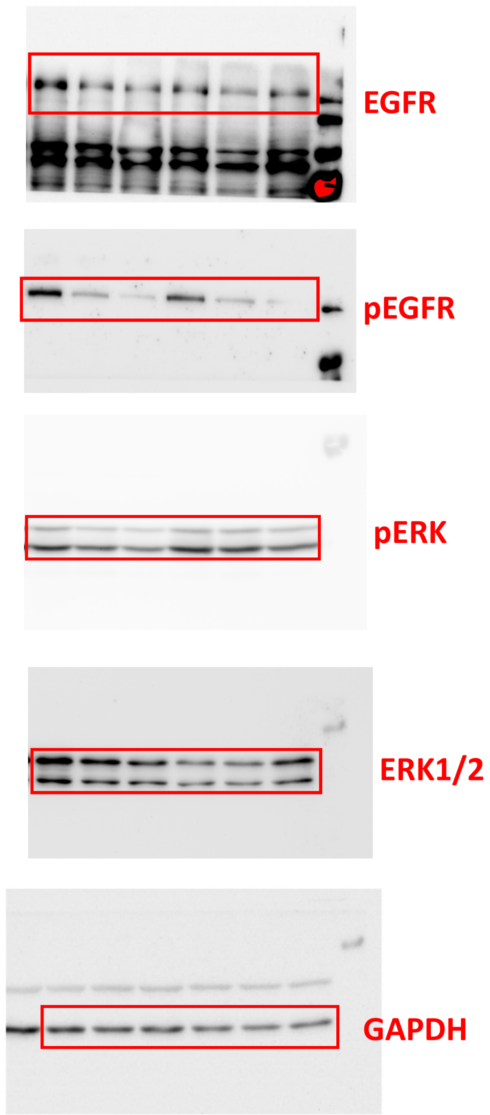

Figure S7a

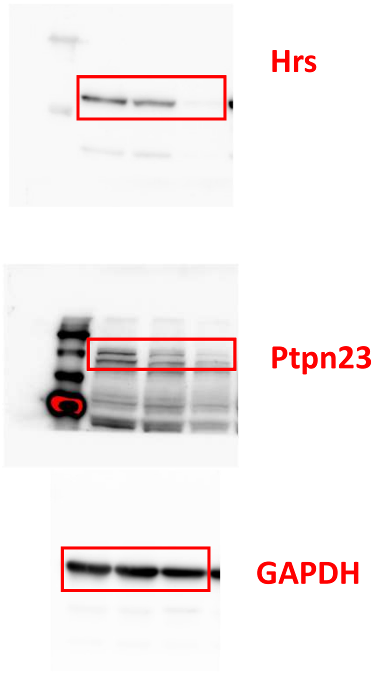

Figure S8a

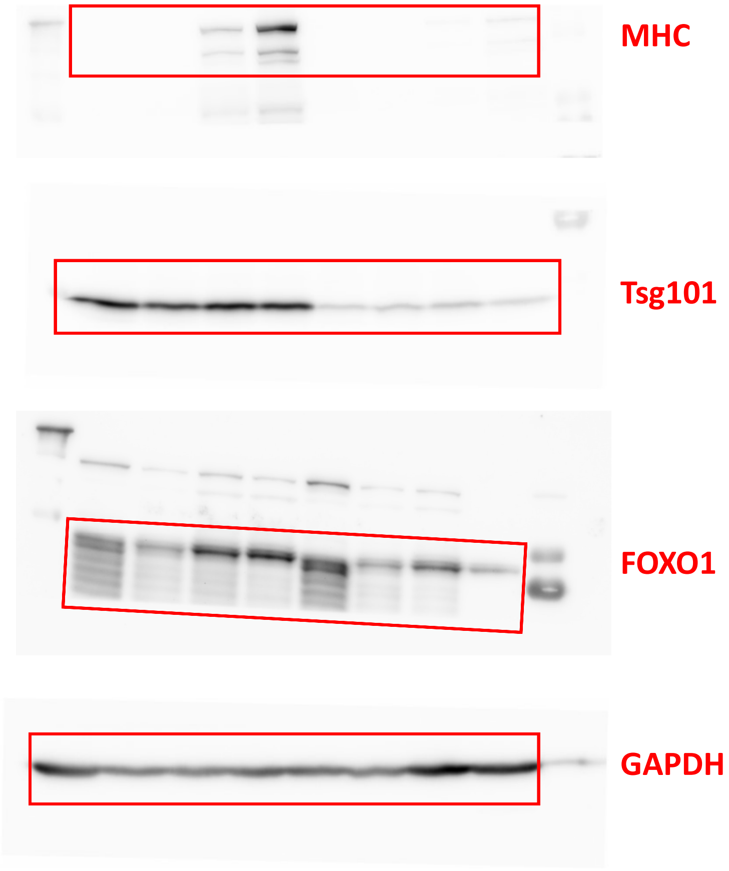

Figure S9a

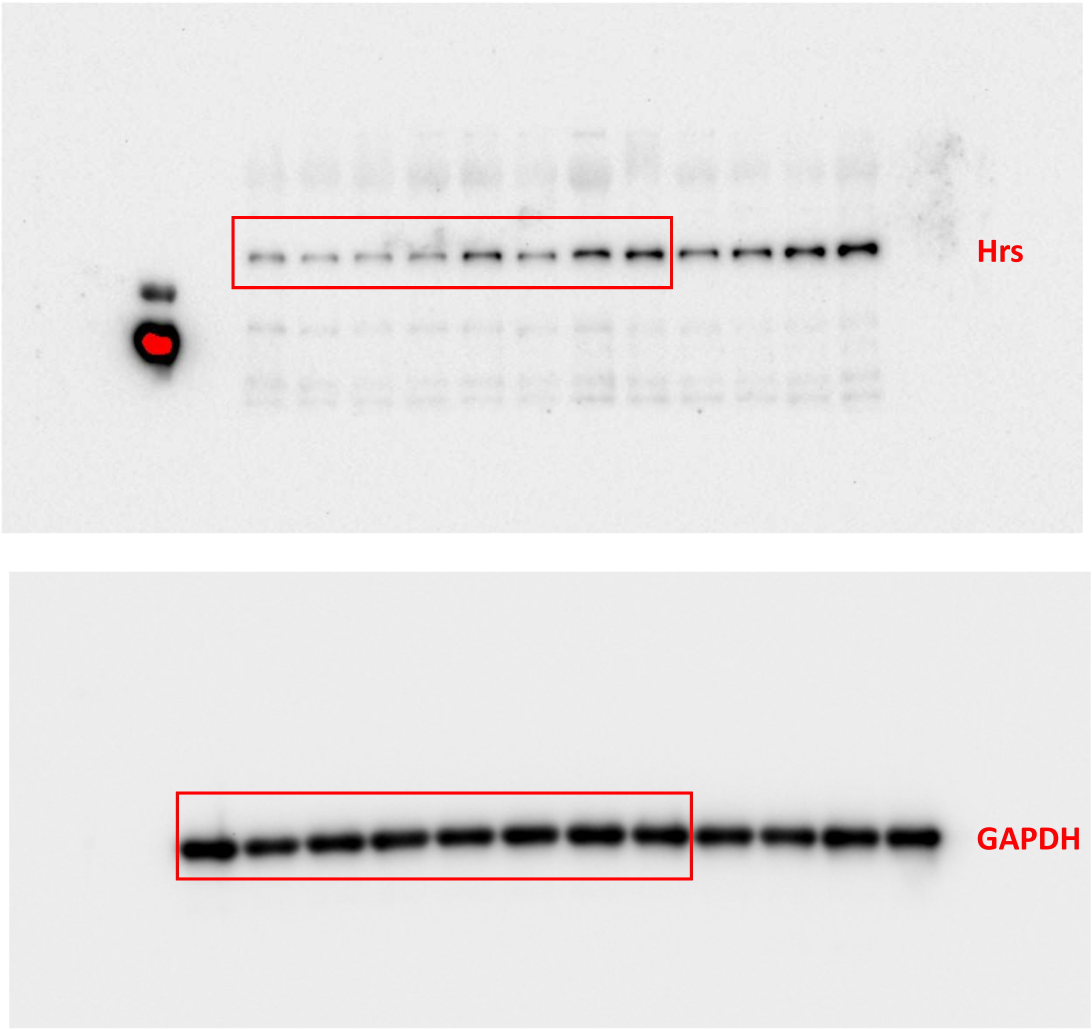

Figure S9c

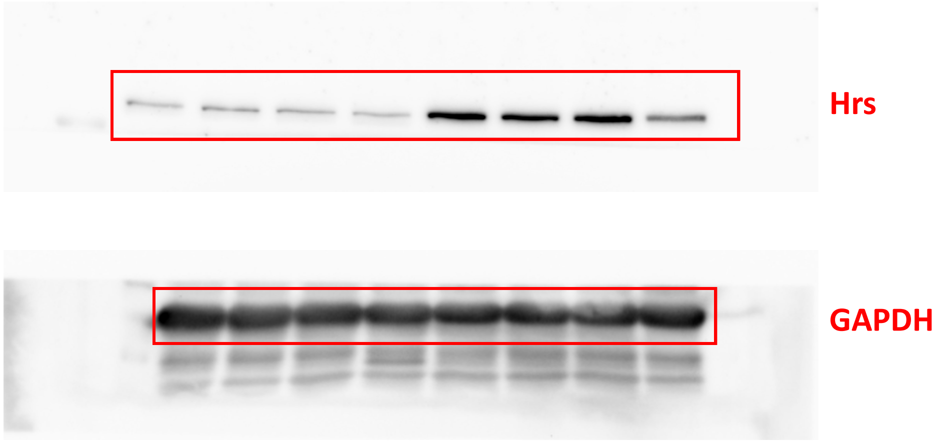

Supplement: Supplementary file 10 — Additional file 10. Raw-data-Western blotting. [file 12915_2021_1091_MOESM10_ESM.pdf]
